# A pipeline combining multiple strategies for prioritizing heterozygous variants for the identification of candidate genes in exome datasets

**DOI:** 10.1186/s40246-017-0107-5

**Published:** 2017-05-22

**Authors:** Teresa Requena, Alvaro Gallego-Martinez, Jose A. Lopez-Escamez

**Affiliations:** 1 Otology & Neurotology Group CTS495, Department of Genomic Medicine, GENYO - Centre for Genomics and Oncological Research – Pfizer/University of Granada/Junta de Andalucía, PTS, 18016 Granada, Spain; 2 Department of Otolaryngology, Complejo Hospitalario Universidad de Granada (CHUGRA), ibs.granada, 18014 Granada, Spain

**Keywords:** Exome sequencing, Variants filtering, Phenotype, Autosomal dominant diseases, Human phenotype ontology, Hearing loss, Meniere disease

## Abstract

**Background:**

The identification of disease-causing variants in autosomal dominant diseases using exome-sequencing data remains a difficult task in small pedigrees. We combined several strategies to improve filtering and prioritizing of heterozygous variants using exome-sequencing datasets in familial Meniere disease: an in-house Pathogenic Variant (PAVAR) score, the Variant Annotation Analysis and Search Tool (VAAST-Phevor), Exomiser-v2, CADD, and FATHMM. We also validated the method by a benchmarking procedure including causal mutations in synthetic exome datasets.

**Results:**

PAVAR and VAAST were able to select the same sets of candidate variants independently of the studied disease. In contrast, Exomiser V2 and VAAST-Phevor had a variable correlation depending on the phenotypic information available for the disease on each family. Nevertheless, all the selected diseases ranked a limited number of concordant variants in the top 10 ranking, using the three systems or other combined algorithm such as CADD or FATHMM.

Benchmarking analyses confirmed that the combination of systems with different approaches improves the prediction of candidate variants compared with the use of a single method. The overall efficiency of combined tools ranges between 68 and 71% in the top 10 ranked variants.

**Conclusions:**

Our pipeline prioritizes a short list of heterozygous variants in exome datasets based on the top 10 concordant variants combining multiple systems.

**Electronic supplementary material:**

The online version of this article (doi:10.1186/s40246-017-0107-5) contains supplementary material, which is available to authorized users.

## Background

Whole-exome sequencing (WES) has become the preferred tool to discover new variants for the diagnosis of genetic diseases, since the protein-coding regions and their boundaries represent only 1.5–2% of the human genome and they accumulate most of the disease-causing mutations: missense and protein-truncating variants (frameshift, splice-acceptor, splice-donor, and nonsense variants) [1, 2]. On average, 45,000 single-nucleotide variants (SNVs) are obtained by WES, 39% are located in coding regions, while 4% are in untranslated regions (UTR), and 56% are in intronic regions near to UTR. In addition, ~90% of SNVs obtained by WES are described in the dbSNP138 based in reference genome (GRCh37 hg19) [3]. However, novel and rare variants (minor allelic frequency (MAF) ≤0.01) identified by WES cannot be interpreted as pathogenic only with this information, and causality must be validated by replication in different individuals with the same phenotype and by functional studies in an appropriate cellular or animal model for each disease. Nevertheless, WES has already shown the efficiency to identify potential disease-causing variants in monogenic diseases [4, 5]. Particularly, WES has been successfully used in rare Mendelian disorders, since most of the disease-causing variants are located in protein-coding regions [5]. Recently, WES studies have been also extended for diagnosis in oligogenic and complex genetic disorders [6–10] and for predicting disease progression [11, 12]. However, when the disease is poorly characterized at the molecular level, the filtering and prioritizing of WES datasets requires a more elaborated search strategy based not only in single variant effects on protein structure or evolutionary conservation but also upon the phenotype description and mathematical interaction models.

The high efficiency of WES data in Mendelian disorders is explained because most of the causal variants in recessive disorders are rare homozygous variants or compound heterozygous variants observed in familiar cases, which are not found in healthy relatives or individuals in the same population [13]. However, the situation is more complex with autosomal dominant (AD) disorders, where a single heterozygous de novo variant can affect the gene function and hundreds of candidate variants need to be filtered. So, an improved workflow to identify potential candidate variants involved in the disease is needed. Software package as MendelScan try to solve this providing a composite score improved with tissue expression data [14]. However, systemic disease or disease involving tissues with multiple cells types and low-quality gene expression data as the cochlea are not easy to analyze with this approach.

Hearing and vestibular disorders are the most common sensory deficits in humans. Hearing loss affect around 5.3% of the world population according to the World Health Organization. Non-syndromic autosomal dominant sensorineural hearing loss (AD-SNHL) remains a challenge for genetic diagnosis, and 33 genes and 60 loci have been involved according to Hereditary Hearing loss Homepage [15], with a considerable overlap in the phenotype and pleiotropy [16].

Meniere’s disease (MD) is clinically defined by episodes of vertigo, tinnitus, and SNHL (MD, [MIM 156000]) [17], and it has a prevalence about 0.5–1/1000 individuals. Most of the patients are considered sporadic, although around 8–10% are familial cases in European descendent population [18–20]. Previous linkage studies in familial MD (FMD) have found candidate loci at 12p12.3 in a large Swedish family [21] and 5q14-15 in another German family [22], but the involved genes were not identified. Recently, WES analyses have identified *DTNA*, *FAM136A*, and *PRKCB* as potential causal genes in FMD [9, 10]. MD is a clinical syndrome, and its phenotype may overlap with different conditions including vestibular migraine or autoimmune inner ear disease [16]. In contrast, other AD diseases with a more precise phenotype, such as Centro Nuclear Myopathy (CNM), an inherited neuromuscular disorder characterized by congenital myopathy with a histopathological diagnosis (centrally placed nuclei on muscle biopsy), have a reduced number of causal variants.

**Fig. 1:**
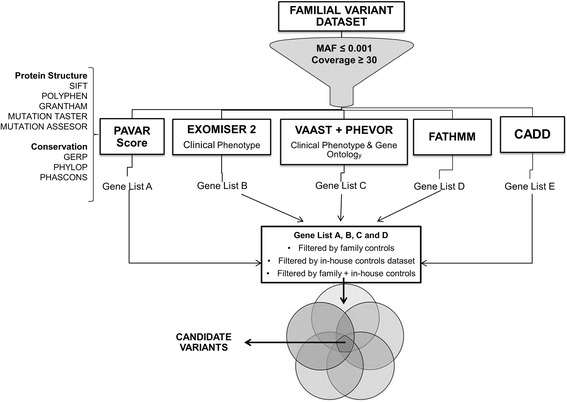
Design of the study. Pipeline: overview of the methods to filter and prioritize WES datasets in autosomal dominant disorders. Datasets were filtered by MAF and Coverage ≥30. Each dataset was analyzed and scored independently by each tool, generating a list of ranked variants or genes. The gene lists obtained were filtered by different control datasets. Finally, the gene lists were merged and yielded a shortlist of variants to be tested experimentally

The aim of this study is to develop a workflow to improve the filtering and prioritizing of candidate variants and genes in AD disorders by using WES data. We focus mainly in AD familial MD, a complex clinical scenario with clinical and genetic heterogeneity, few cases per family, incomplete penetrance, and variable expressivity [23, 24]. The pipeline proposed is based on (1) the combination of several tools to score variants according to its effect on protein structure and phylogenetic conservation, (2) the ranking according to available information on phenotype databases, (3) the comparison with two integrated systems (CADD and FATHMM), and (4) the use of un-affected relatives as control to filter candidate variants. The pipeline is summarized in Fig. 1.

## Results

Six prioritizing systems were selected and combined in the pipeline to filter and rank rare variants in exome sequencing data. Two of them were based upon protein structure and sequence conservation across species: (a) an in-house Pathogenic Variant (PAVAR) score and (b) the Variant Annotation Analysis and Search Tool (VAAST) [25], and the other two prioritize according to the Phenotype Ontology information: (c) Exomiser v2 [26] and (d) VAAST-Phevor [27]. And finally two integrated tools were compared and added to the system CADD [28] and FATHMM [29].

### Comparison of prioritizing strategies with FMD exome datasets

Table 1 shows the number of variants obtained for each FMD dataset with the six systems after filtering by several control datasets. We included the number of ranked variants with enough score to be prioritized, according to each of the six systems (thresholds are described in the “Material and methods” section). Mean values obtained for each family dataset were highly variable for each system, and they were dependent on the number of cases and controls available for each family.

**Table 1:** Number of remaining variants per family dataset according to the filtering strategy

Family dataset	FMD exomes (*N*)	Control dataset (*N*)	PAVAR score ≥5 (*N*)	Exomiser score ≥1.46 × 10^−5^ (*N*)	VAAST (*p* value ≤1)	VAAST-Phevor (*p* value ≤1)	CADD score ≥15 (*N*)	FATHMM score ≤−1.5 (*N*)
1	3	F (1)	17 (134)	308 (1437)	40	39	15 (38)	7 (35)
		T-F (29)	15 (106)	78 (296)	48	44	18 (36)	7 (34)
		T (30)	10 (68)	42 (175)	27	27	12 (25)	5 (23)
2	2	F (3)	4 (58)	60 (270)	53	22	9 (18)	1 (14)
		T-F (27)	9 (73)	89 (369)	146	135	12 (28)	1 (25)
		T (30)	2 (34)	9 (39)	19	16	5 (13)	0 (11)
3	3	F (2)	9 (68)	151 (862)	23	23	9 (20)	1 (14)
		T-F (28)	13 (92)	67 (309)	38	38	17 (25)	5 (20)
		T (30)	6 (32)	24 (104)	16	16	7 (10)	1 (7)
4	3	F (0)	31 (283)	394 (2198)	54	46	34 (90)	4 (86)
		T (30)	4 (34)	20 (72)	19	17	5 (14)	1 (14)
5	3	F (3)	16 (83)	93 (391)	68	22	7 (20)	1 (15)
		T-F (27)	14 (113)	89 (430)	52	45	14 (35)	7 (28)
		T (30)	5 (36)	18 (67)	11	9	4 (9)	1 (6)
Mean (1–5)	21	F	15.4 ± 10.21 (125)	251.5 ± 143.83 (1032)	47 ± 16.95	30.4 ± 11.33	14.8 ± 9.96	2.8 ± 2.4
		T-F	12.75 ± 2.63 (96)	85 ± 28.66 (351)	71 ± 50.35	65.5 ± 46.44	13.5 ± 4.38	5.0 ± 2.44
		T	5.2 ± 2.97 (51)	31 ± 13.94 (155)	28.2 ± 5.81	5.81 ± 6.44	6.60 ± 2.87	1.6 ± 1.74

All variants with a MAF >0.001 were discarded. Setting for each software threshold is described in the “Material and methods” section

*p* values for VAAST and Phevor were not corrected since they were used as thresholds according to the user’s guide

*F* family controls exome dataset, *T-F* in-house controls exome dataset without family control dataset, *T* in-house and family control datasets

**Fig. 2:**
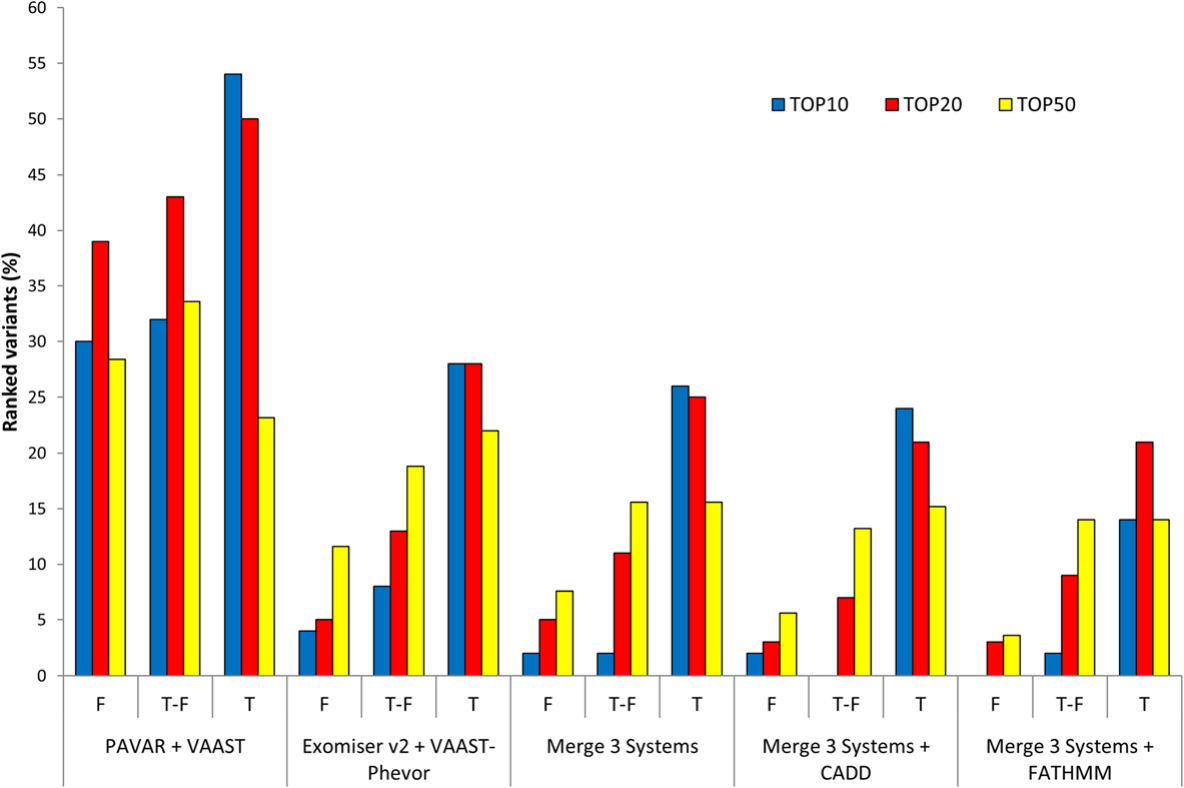
Prioritized variants in FMD datasets. Percentage of the variants ranked and shared in top 10 (*blue*), 20 (*red*), and 50 (*yellow*) ranked variants by (a) PAVAR score and VAAST; (b) Exomiser v2 score and Phevor; (c**)** the combination of the three systems (PAVAR, Exomiser v2, VAAST, VAAST-Phevor); (d) the combination of the three systems (PAVAR, Exomiser v2, VAAST, VAAST-Phevor) and CADD; and (e) the combination of the three systems (PAVAR, Exomiser v2, VAAST, VAAST-Phevor) and FATHMM. F = family controls exome dataset, T-F = in-house controls exome dataset without family control datasets, and T = in-house and family control datasets

We selected the top 10, 20, and 50 ranked variants from each prioritizing system and filtered them using the different control datasets (F, T-F, and T) to analyze the concordance between methods. Figure 2 shows the concordance between all systems. Although PAVAR score and VAAST use a different methodology, both systems show the highest concordance rate to filter and prioritize the candidate variants. Between 20 and 55% of ranked variants were matched in top 10, top 20, and top 50. However, the observed variability in the ranked variants between the different systems is caused by the control datasets (F, T-F, or T) used to filter the variants. In contrast, Exomiser v2 and VAAST-Phevor prioritized according to the Phenotype Ontology information (HPO term) [30], but the maximum correlation between systems was 28% when the largest control dataset (T) was used to filter. Therefore, only the variants located in genes previously associated with the phenotype were matched by different systems. Consequently, the combinations of PAVAR, VAAST-Phevor, and Exomiser v2 only matched in few variants (2–26%), which were top ranked and highly related with MD HPO terms. A similar concordance was obtained between the combination of that three and other combined systems as CADD or FATHMM.

The maximum correlation between CADD and the merge of three systems was 24% in top 10, whereas for FATHMM was 21% in top 20. In both cases, this correlation was obtained after using the largest controls’ dataset (T) to filter the variants.

### Benchmark in exome datasets containing variants described in AD-SNHL and CNM genes

We compared the ability of these variant prioritizing tools to identify AD variants in small familial exome data files by a benchmarking procedure. Since the structure of the families as well as the number of cases and controls available for each pedigree could generate a bias in the benchmarking analyses, multiple families were tested.

**Fig. 3:**
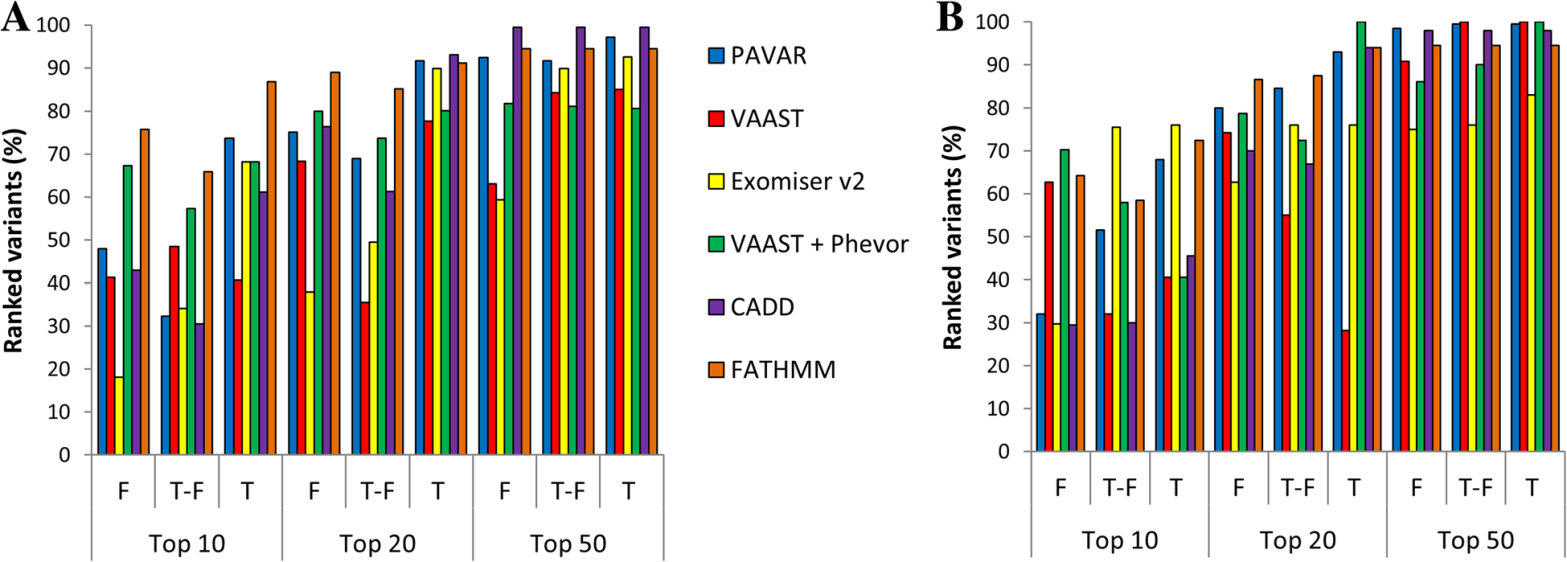
Benchmarking analyses for PAVAR (*blue*), VAAST (*red*), Exomiser v2 (*yellow*), VAAST-Phevor (*green*), CADD (*purple*), and FATHMM (*orange*). Bar charts show the percentage of hearing loss (**a**) and CNM (**b**) variants ranked by each strategy among the top 10, 20, or 50, after filtering by each control dataset filter. F = family controls exome dataset, T-F = in-house control data exome dataset without family control datasets, and T = in-house and family control datasets

Figure 3 shows the percentage of ranked variants in top 10, 20, and 50 by the six systems for both, hearing loss variants (Fig. 3a) and CNM variants (Fig. 3b). In top 10 and 20, the observed percentages were highly variable between each system, particularly depending on the control dataset used.

**Fig. 4:**
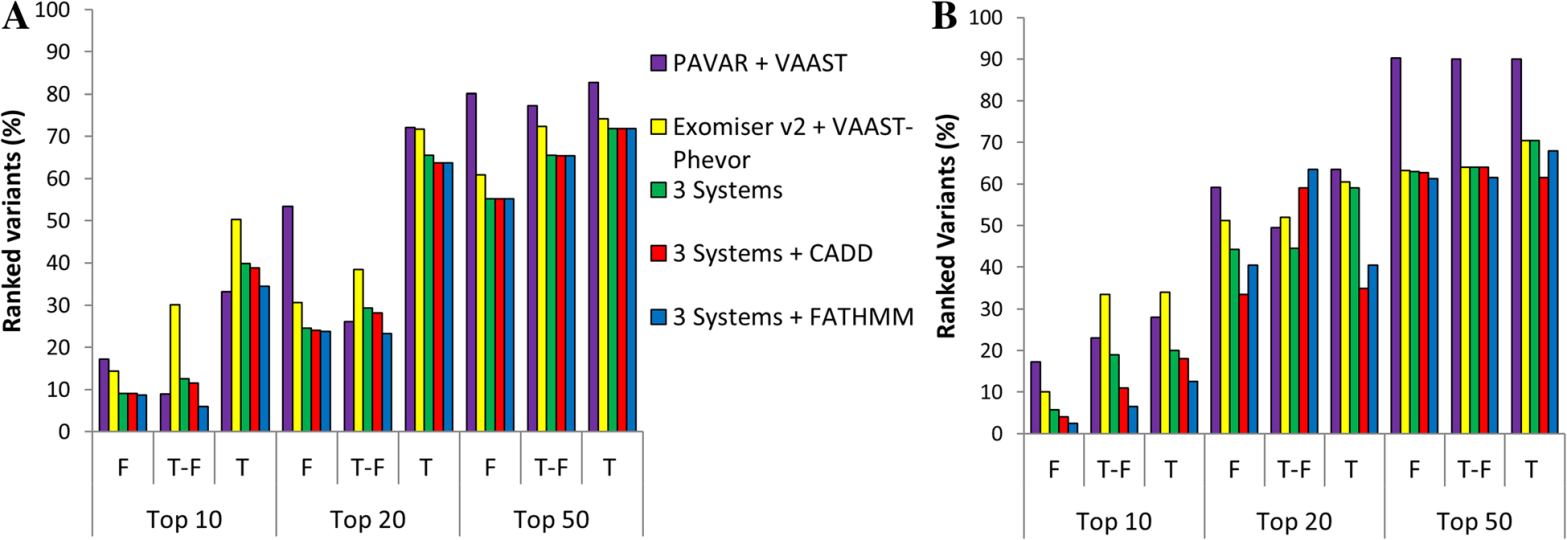
Benchmarking analyses combining prioritizing strategies. Bar charts show the percentage of shared variants for hearing loss (**a**) and CNM (**b**), ranked by PAVAR + VAAST (*purple*), Exomiser v2 + (VAAST-Phevor) (*yellow*), the three systems (*green*) the three systems and CADD (*red*), and the three systems and FATHMM (*blue*) among the top 10, 20, or 50, after filtering by different control datasets. F = family controls exome dataset, T-F = in-house control data exome dataset without family control dataset, and T = in-house and family control datasets

Next, we selected the top 10, 20, and 50 ranked variants from each prioritizing system and filtered them for the different datasets (F, T-F, and T) to analyze the concordance between the different methods. Figure 4 illustrates a progressive increase of concordance between systems in the top 10, 20, and 50 ranked variants for both disorders. Exomiser v2 and VAAST-Phevor yielded higher correlations in the top 10 and 20, highlighting that both tools identify similar genes associated with the HPO term for a given phenotype. This pattern was more prominent in top 10 ranked variants for AD-SNHL datasets in the benchmarking, reaching a 50% of concordance (Fig. 4a), whereas in CNM datasets, only 34% of concordance was found (Fig. 4b). In contrast, low correlations were obtained between PAVAR score and VAAST (9–33%), mainly in the top 10 ranked, means that few variants are considered as candidates by both systems as real pathogenic variants. As a result, potentially pathogenic variants located in genes with HPO terms associated with the disease were shared by PAVAR, Exomiser v2, and VAAST-Phevor and tending to be ranked in the top 10.

A similar percentage was obtained when we add CADD to the combined system. However, the combination of multiple systems with CADD did not reduce the list of candidate variants in the top 10 ranking.

**Fig. 5:**
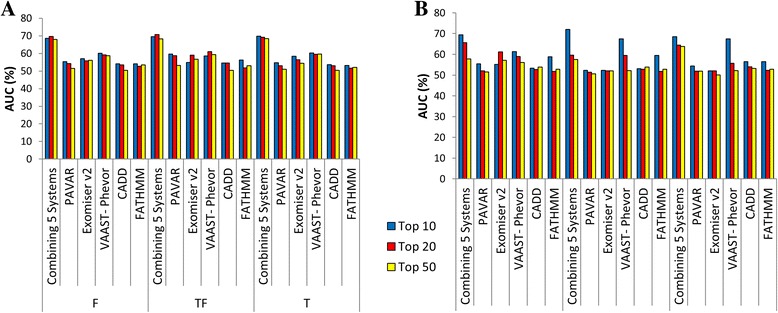
Precision and accuracy of the different systems was estimated by calculating the AUC. Top 10 (*blue*), top 20 (*red*), top 50 (*yellow*). Bar charts show AUC percentages to identify real pathogenic variants for hearing loss (**a**) and CNM (**b**) among the *top* 10, 20, or 50 ranked variants and according to control dataset filter. F = family controls exome dataset, T-F = in-house control data exome dataset without family control datasets, and T = in-house and family control datasets

Next, 200 variants were randomly selected for each disease to build synthetic datasets. So, 42% for AD-SNHL and 25.5% CNM were previously described in HGDB as pathogenic (Additional file 1: Table S1 and S2). So, multiple logit regression models were performed to assess the accuracy to predict correctly candidate variants associated with each phenotype. The area under the curve (AUC) for each system was calculated to assess the precision and accuracy to identify candidate variants for both diseases in several families (Additional file 1: Table S3). On average, the combination of PAVAR, Exomiser v2, VAAST-Phevor, CADD, and FATHMM predicts potentially pathogenic variants associated with the phenotype between 68 and 71% of times in top 10, for both diseases (Fig. 5a, b). These results were statistically significantly better than any single method (*p* values shown in Additional file 1: Table S3).

## Discussion

The combination of linkage analysis and WES in large multicase pedigrees has shown a high effectiveness to identify disease-causing variants in rare Mendelian disorders [4, 5]. However, small pedigrees with a few available cases are the most common clinical scenario and a challenge for the genetic diagnosis of dominant disorders, mainly those with overlapping phenotypes or incomplete penetrance such as AD-SNHL [31, 32], CNM [33], and MD [20]. Despite the increasing number of bioinformatics tools to analyze WES data [34, 35], the list of genes that must be experimentally validated for these diseases is too large.

The first issue to resolve for variant identification is the alignment of reads and variant calling algorithms. Current approaches have developed pipelines that combine tools to obtain consistent identification of variant and facilitate the process [36, 37]. However, these pipelines do not provide functional annotation. Other pipelines go further, and they implement user-friendly graphic interface and include Annovar-based functional annotation [38]. However, our results show that the combination of multiple bioinformatics tools is a reliable strategy to reduce the list of candidate variants and to facilitate the identification of the disease-causing variants in small pedigrees. These results are consistent with previous studies designed to improve the yield of several prioritizing tools [39, 40].

The list of candidate variants generated by each system is usually too large to be validated experimentally (Table 1). So, the most common strategy is to filter by familiar controls to eliminate private familial variants and by controls’ dataset from the same population to eliminate population-specific variants. However, the clinical evidence of incomplete penetrance or late age of onset of the disease should exclude the use of familial control datasets. Our results show that by combining five tools (PAVAR, Exomiser v2, VAAST-Phevor, CADD, FATHMM), the list of candidate variants is reduced and this facilitates the identification of potential disease-causing variants (Fig. 5).

Discrepancies between all the prioritization systems evaluated (PAVAR, VAAST, Exomiser v2, VAAST-Phevor, CADD, FATHMM) were found in the ranked results for all the diseases tested (Table 1 and Fig. 3). Consequently, systems based on the same criteria, protein structure, and sequence conservation or Phenotype Ontology information, were clustered to analyze the concordance between them in the top 10, 20, and 50 ranked variants. Although PAVAR and VAAST use a different methodology, both prioritize variants according to the intrinsic effect on the protein of the variants. Of note, MD, AD-SNHL, and CNM showed similar correlation scores between PAVAR and VAAST for top 10 and 20 ranked variants. Both systems were more concordant when in-house control datasets or the merge of in-house and family control datasets were used to filter. Although familial controls are important to filter private variants, a large control dataset of the same population is more effective to reduce the list of candidate variants list.

In contrast, the concordance between VAAST-Phevor and Exomiser v2 varies depending on the disease studied. Although both systems are based on phenotype, VAAST-Phevor has a balanced score between potential pathogenicity and the association with the phenotype whereas Exomiser v2 assigns more weight to the phenotype than the potential pathogenicity. Diseases with a well-characterized phenotype by several HPO terms or diseases with known involved genes show a high correlation between VAAST-Phevor and Exomiser v2, as our results confirm for AD-SNHL and CNM. However, since MD only has few HPO terms and no gene associated in public databases, our data show a reduced concordance. In particular, our results show that the correlation between both systems in well diseases with many HPO terms is twice than in disorders with limited phenotypic information such as diseases of the ear for all top 10, 20, and 50 ranked variants. Nevertheless, a high concordance between both systems does not indicate that those variants selected are really disease-causing variants. The degree of concordance between both systems only demonstrates that the candidate genes are associated with the phenotype, but not necessarily its pathogenicity.

Initially, our pipeline joins both approaches by the identification of variants ranked as potentially pathogenic by the PAVAR score and associated them with the phenotype by both Exomiser v2 and VAAST-Phevor. The combination of the three strategies gives few variants ranked in the top 10 or 20 and produces a short list of candidate variants to be validated experimentally [9, 10, 41]. In addition, other combined systems were added and the list was reduced. Logit regression models and benchmarking analyses show that the combination of PAVAR, Exomiser v2, VAAST-Phevor CADD, and FATHMM not only reduced the list of candidate variants to be validated; this combined approach is more efficient to predict potential diseases-causing variants than each system separately. This enhanced efficiency is observed independently of the type of control datasets used. Our results confirm previous studies showing that prioritizing tools have less ability to rank variants in disorders with no previously known candidate gene [42]. In addition, we demonstrate that the addition of more HPO terms improves the ranking of candidate genes. So, our pipeline allows to obtain a reduced list of variants when incomplete penetrance is found and familial control datasets cannot be used.

This combined strategy has a major limitation: a reduced phenotypic characterization of AD disorders (such as AD-SNHL or MD) will decrease the precision of the pipeline. So, a deep phenotyping and updating of HPO terms in major databases will improve the yield of the system. Although HPO project has been updated in 2017, ear diseases and, particularly, vestibular disorders still have a limited phenotype vocabulary and disease-phenotype annotations [43]. In addition, further improvements in the pipeline should be needed to include structural variants such as frameshift (insertions and deletions), synonymous variants, and copy number variants.

## Conclusion

These results demonstrate that our pipeline combining multiple variant-prioritization algorithms is useful in small family-based analyses. We also showed that the model can reduce the number of variants in synthetic exome datasets with incomplete phenotypes without using familial controls. This approach will be useful when controls are not available or when incomplete penetrance is observed.

## Material and methods

### Patients

Four Spanish AD families with at least two patients with definite MD and a fifth family with monozygotic twins with MD, according to the diagnostic criteria of the Barany Society for familial MD [17], were selected for this study. The clinical phenotype and the pattern of inheritance in these families and their pedigrees were previously reported [10, 20, 41]. The number of asymptomatic relatives selected for WES in each family depended upon two criteria: (a) size and structure of the family, since some families showed patients with incomplete phenotype (i.e., SNHL without episodic vertigo), and (b) the availability to obtain samples from older asymptomatic relatives, which could be used as controls. All the procedures described were performed in accordance with the highest ethical standards on human experimentation, the Helsinki Declaration of 1975 and the EU regulations on biomedical research. In addition, this study was approved by the Review Board for Clinical Research of Instituto Biosanitario de Granada, and a written informed consent to donor biological samples was obtained from all subjects.

### Whole exome sequencing (WES)

DNA was isolated from peripheral blood samples as previously described [9, 10] Exons and flanking intron regions were captured according to the methods previously described [9, 10]. Library products were sequenced with SOLiD 5500xl platform with Exact Call Chemistry and 200× of sequencing depth. A mean of 50–60 million of reads were obtained per sample. The quality of the reads was analyzed with SAMtools [44], MAQtools [45], and FastQC software (Babraham Bioinformatics), and shorter reads (<25) as well as all duplicate reads were deleted. The reads were aligned with the reference genome (GRCh37 hg19) with Bioscope™ (Applied Biosystems, Foster City, CA, USA) using the default settings. Results from Bioscope™ were filtered by depth >30 reads [46] and quality of the assigned genotype ≥100. This analysis identified SNVs, copy number variants, and frameshift variants (insertion and deletions). However, we only considered SNVs for this study.

### Bioinformatics analysis

For each family, heterozygous SNVs found in all the affected cases with complete phenotype of the family were selected. The 1000 genome project [47], ExaAC database [48], and Exome Variant Server (EVS) were used to annotate the MAF and function for each variant (Additional file 1: Table S4). All SNVs were filtered by MAF. For MD and AD-SNHL, variants with MAF ≥0.001 were discarded, since MD has a prevalence of 10–225 cases/100,000 individuals [49, 50] and the low prevalence described for AD-SNHL [51]. For CNM, variants with MAF ≥0.0001 were also discarded, since CNM is considered as a rare disease with a very low prevalence (1/25,000 males).

The pipeline was designed using different strategies to filter and prioritize SNVs: (a) the calculation of a pathogenic variant (PAVAR) risk composite score; (b) Exomiser v2 software [26]; (c) VAAST annotation tool [25]; and (d) a combination of VAAST and Phevor tools [27]. However, Phevor returns the same results than VAAST, but ranked by phenotype. In addition, other composite algorithms were used CADD [28] and FATHMM [29]. So, the shared candidate variants were selected. All variants were considered as potentially pathogenic according to the ACMG Standards and Guidelines [52], and all digital resources used are listed in Additional file 1: Table S5.

In some AD diseases, incomplete penetrance was found; subsequently, familial controls could not be used to filter variants. Different control datasets collected for previous projects were used to evaluate the efficiency of our pipeline despite of the observed incomplete penetrance. F = family controls exome dataset, T-F = in-house control data exome dataset without familial control datasets, and T = in-house and family control datasets.

- Pathogenic variant risk composite score (PAVAR score)
  Functional annotation was used to prioritize SNVs, according to the effect on protein structure and phylogenetic conservation. Sequence conservation across species is a major criterium to assess the variant, and the number of compared species varies according to the tool. To estimate the risk of a SNV to become a pathogenic variant, we used a seven-point scoring system based upon open-access prediction bioinformatics tools. ANNOVAR and SeattleSeq Annotation tools were used to achieve the score of SIFT (Sort Intolerant from Tolerant) [53], PolyPhen2 (Polymorphism Phenotyping v2) [54], Grantham’s Matrix [55], GERP++ (Genomic Evolutionary Rate Profiling) [56], Mutation taster [57], PhastCons, and PhyloP [58]. The threshold to consider each variant as pathogenic is described in Additional file 1: Table S6, according to the default settings suggested for each software developer. PAVAR score is calculated as the sum of the score obtained by seven systems. Each system adds one point if the variant is considered as potentially damaging and zero if it is benign. So, the higher the score is, the high the risk of pathogenicity for a given variant. PAVAR score cannot be calculated for nonsense variants, since protein structure tools cannot assign any value. Since nonsense variants can modify dramatically the sequence of the protein, they were considered directly as the maximum PAVAR score = 7. All the variants with a score ≥5 were not filtered, and they were considered as candidate variants.
- Exomiser v2 software
  Exomiser v2 prioritizes SNVs by comparing the phenotype across species, according to the inheritance pattern, using the mouse and fish as a model organism phenotype [26]. Variant Call Format (VCF) files were analyzed with the following parameters: (a) HPO terms, Vertigo (HP:0002321), Tinnitus (HP:0000360), and Hearing Impairment (HP:0000365), were selected for Clinical Phenotype and (b) AD inheritance model. Since there are only three HPO terms associated with MD according to the public Human Phenotype Ontology database, but no gene is still included on it, the “Exomiser Gene Combined Score” generated very low values. So, variants with a threshold ≥1.46 × 10^−5^ were considered as candidate variants. Exomiser v2 allows the use of several HPO terms, but Phevor only allows five HPO terms. To compare both systems, only five HPO terms were selected for the benchmarking analyses. The five HPO terms most commonly associated with each disease were selected (Additional file 1: Table S7 and S8).
- VAAST annotation tool
  The third approach was to annotate and filter SNVs, according to the dominant inheritance pattern by VAAST software [25]. All case and control VCF files were processed according to the manual provided in the official website. Case files from the same pedigree were combined by the VAAST selection tool (VST) into a single condenser file; SNVs found in all the affected cases were selected. The quality of the resulting files was measured using the background provided: 1KGv3_CG_Div_NHLBI_dbSNP_RefSeq.cdr. A *p* value >0.05 indicates that there is no significant difference between the files (Additional file 1: Table S9). The next step was to search for candidate genes and their potential disease-causing variants. Each family dataset was filtered with the following parameters: (a) dominant inheritance, (b) incomplete penetrance, (c) maximum combined population frequency for the disease-causing alleles >0.0005 [51], and (d) 1 × 10^6^ permutations per analysis to achieve a significant *p* value after Bonferroni correction. Variants with an alpha error ≤1 were considered as possibly pathogenic.
- Phevor tool
  In the fourth approach, the list of the resulting genes generated by VAAST tool was uploaded to the Phevor Webtool (phenotype driven variant ontological re-ranking tool) to prioritize candidate genes, according to phenotype and HPO terms [30]. To run the analyses for MD, AD-SNHL, and CNM, the phenotypes were generated in Phevor using HPO term described in Additional file 1: Tables S7 and S8. Exomiser v2 only admits HPO term so to compare with Phevor; Disease Ontology Terms and Gene Ontology Terms were not used. No threshold value was applied in these analyses since the list of variants is generated from pre-filtered variants from VAAST.
- Combined Annotation-Dependent Depletion (CADD)
  CADD v1.3 [28, 59] is pre-computed score database that is based on classifier algorithms. The major goal of CADD is to predict the deleterious, functionally significant and pathogenic variants from diversified class of variants by integrative annotations. For each variant, CADD generates the combined annotation score (c-score) as an output and all scores were referenced against the pre-computed c-scores of 8.6 billion possible human SNPs. In CADD scoring criteria, functional variants should possess c-score greater than or equal to 10, whereas damaging variants show the c-score greater than or equal to 20 and the most lethal human variants show the c-score of greater than or equal to 30. To identify causal variants, a score ≥15 was considered as potentially pathogenic.
- Functional Analysis through Hidden Markov Models (FATHMM)
  FATHMM [29] predict the functional effects of protein missense mutations by combining sequence conservation within hidden Markov models (HMMs), representing the alignment of homologous sequences and conserved protein domains, with “pathogenicity weights”, representing the overall tolerance of the protein/domain to mutations. The prediction outputs are scored, and the majority of disease-associated AASs fell below −3 and −1.5 threshold. To identify potential causal variants, a score ≤−1.5 was considered as potentially pathogenic.

### Benchmarking procedures

The efficiency of the workflow was tested by benchmarking procedures in different synthetic family datasets with MD. In addition, a group of no familial healthy controls was tested to identify any bias caused for MD that could influence in the analysis. Moreover, two AD disorders were selected: (a) autosomal dominant sensorineural hearing loss (AD-SNHL) and (b) Central nuclear myopathy (CNM). AD-SNHL has 33 genes diseases, but the phenotype could overlap with MD. To avoid the bias of analyzing AD-SNHL and MD, we selected another disease (CNM) with no overlap in the phenotype with MD. CNM was selected because it has five different genes to perform the benchmarking analysis. The best characterized genes available for AD-SNHL included in the Hereditary Hearing Loss Homepage and CNM genes described in Orphanet were selected (Additional file 1: Table S5). For these genes, exome sequencing data of all exonic variants, in VCF format, were obtained from the public ESP database. Next, 200 variants for each disease were randomly selected to perform benchmarking analyses, but we also checked that at least part of them were described as pathogenic or associated with the disease in human mutation database (HGMD) (Additional file 1: Table S1 and S2). To perform the analyses, the synthetic files were built inserting two random variants into real cases VCF files of each family. These synthetic family files for both diseases were analyzed with the six systems. The top 10, 20, and 50 ranked variants for AD-SNHL and CNM were analyzed by each separate system and by all combined strategies.

### Statistical analysis

Logit regression model was built to assess the accuracy to predict correctly pathogenic variants associated with the phenotype. Firstly, variants selected for benchmarking analysis were classified as pathogenic or benign according to HGMD. The ranks conferred by each system were converted into ranks predictor-wise and normalized in [0, 1], according to top 10, 20, or 50. ROC curves were generated to determine the ability to predict real causal variants based on models consisting of the combination of the five systems (PAVAR, Exomiser v2, VAAST-Phevor, CADD, and FATHMM) and each individual system. In all the cases, the analyses were performed for the top 10, 20, and 50 ranked variants and using different control datasets to filter for private variants. AUCs were calculated for each ROC curves (Additional file 1: Table S3). The statistical differences between AUCs were calculated by analysis of variance. The logit regression models obtained, according to the different combinations and ROC curves, were analyzed with R version 3.0.3 and RStudio version 0.98.1102.

## Additional file

Additional file 1: Additional Tables for Requena et al., “A pipeline combining multiple strategies for prioritizing heterozygous variants for the identification of candidate genes in exome datasets”. **Table S1** Two hundred randomly selected SNV located in genes causing autosomal dominant sensorineural hearing loss. **Table S2** Two hundred randomly selected SNV located in genes causing Centro Nuclear Myopathy. **Table S3** Logit regression model to predict pathogenic variants is based on models consisting of single or multiple prediction tools for the top 10, 20, and 50 ranked variants for each tool, respectively. **Table S4** Number of SNV obtained in 21 exome datasets according to its effect on protein sequence and position on the reference genome (GRCh37 hg19). **Table S5** Web Resources, the URLs for software presented. **Table S6** Pathogenic Variants Scoring System (PAVAR). **Table S7** HPO terms used to describe the AD-SNHLs. **Table S8** HPO terms used to describe the CNMs. **Table S9** VAAST files. *p* value of quality, no significant differences were found between WES data and the background. (DOCX 180 kb)

## Abbreviations

AD: Autosomical dominant
AD-SNH: Non-syndromic autosomal dominant sensorineural hearing loss
CADD: Combined annotation-dependent depletion
CNM: Centro Nuclear Myopathy
FATHMM: Functional analysis through hidden markov models
FMD: Familial MD
HPO term: Human Phenotype Ontology term
MAF: Minor allelic frequency
MD: Meniere’s disease
PAVAR: Pathogenic variant risk composite score
SNV: Single-nucleotide variants
UTR: Untranslated regions
VAAST: Variant annotation analysis and search tool
WES: Whole-exome sequencing
