# Supplementary material for: A pipeline combining multiple strategies for prioritizing heterozygous variants for the identification of candidate genes in exome datasets
Source: Hum Genomics. 2017 May 22;11:11. doi: 10.1186/s40246-017-0107-5 (PMC5441048; doi:10.1186/s40246-017-0107-5)
Supplement: Additional file 1: — Additional Tables for Requena et al., “A pipeline combining multiple strategies for prioritizing heterozygous variants for the identification of candidate genes in exome datasets”. Table S1 Two hundred randomly selected SNV located in genes causing autosomal dominant sensorineural hearing loss. Table S2 Two hundred randomly selected SNV located in genes causing Centro Nuclear Myopathy. Table S3 Logit regression model to predict pathogenic variants is based on models consisting of single or multiple prediction tools for the top 10, 20, and 50 ranked variants for each tool, respectively. Table S4 Number of SNV obtained in 21 exome datasets according to its effect on protein sequence and position on the reference genome (GRCh37 hg19). Table S5 Web Resources, the URLs for software presented. Table S6 Pathogenic Variants Scoring System (PAVAR). Table S7 HPO terms used to describe the AD-SNHLs. Table S8 HPO terms used to describe the CNMs. Table S9 VAAST files. p value of quality, no significant differences were found between WES data and the background. (DOCX 180 kb) [file 40246_2017_107_MOESM1_ESM.docx]

**A pipeline combining multiple strategies for prioritizing heterozygous variants for the identification of candidate genes in exome datasets**

**Teresa Requena**^1*^**, Alvaro Gallego-Martinez**^1^**, Jose A Lopez-Escamez**^1,2^

^1^Otology & Neurotology Group CTS495, Department of Genomic Medicine, GENYO - Centre for Genomics and Oncological Research – Pfizer/University of Granada/ Junta de Andalucía, PTS, Granada, 18016 Spain.

^2^ Department of Otolaryngology, Complejo Hospitalario Universidad de Granada (CHUGRA), ibs.granada, Granada, 18014 Spain

* Corresponding author: mariateresa.requena@genyo.es

**Table S1** Two hundred randomly selected SNV located in genes causing autosomal dominant sensorineural hearing loss.

| CHR. | POSITION | Rs | REF | ALT | FUNTION | GEN | MAF | HMDB | PHENOTYPE MIM NUMBER | DISEASE |
| --- | --- | --- | --- | --- | --- | --- | --- | --- | --- | --- |
| 1 | 35250451 | rs373725070 | G | A | missense | GJB3 | 8.245E-06 | yes |  |  |
| 1 | 35250457 | rs1805063 | C | T | missense | GJB3 | 0.0242 | yes |  |  |
| 1 | 35250652 | rs140829910 | T | C | missense | GJB3 | 8.242E-06 |  |  |  |
| 1 | 35250673 | rs375681439 | C | T | missense | GJB3 | 0.00004946 |  | 612644 | DFNA2B |
| 1 | 35250862 | rs376748531 | G | A | missense | GJB3 | 0.0003 |  |  |  |
| 1 | 35250892 | rs80297119 | T | G | missense | GJB3 | 0.0016 | yes |  |  |
| 1 | 35250910 | rs74315318 | G | A | missense | GJB3 | 0.0005 | yes |  |  |
| 1 | 35251030 | rs373815705 | C | A | missense | GJB3 | 0.0004 | yes |  |  |
| 1 | 41296788 | rs142453905 | T | C | missense | KCNQ4 | 0.0005 |  |  |  |
| 1 | 41296828 | rs34287852 | T | G | missense | KCNQ4 | 0.2028 | yes | 600101 | DFNA2A |
| 1 | 41300706 | rs370248473 | G | A | missense | KCNQ4 | 0 |  |  |  |
| 3 | 191098660 | rs114502673 | A | G | missense | CCDC50 | 0.0065 |  | 607453 | DFNA44 |
| 4 | 6279336 | rs111773340 | C | A | missense | WFS1 | 0.0002 |  |  |  |
| 4 | 6290774 | rs145639028 | G | A | missense | WFS1 | 0.00004427 | yes |  |  |
| 4 | 6290847 | rs113651985 | C | T | missense | WFS1 | 0.0005 |  |  |  |
| 4 | 6292945 | rs115346085 | G | A | missense | WFS1 | 1 | yes |  |  |
| 4 | 6293040 | rs41264699 | A | C | missense | WFS1 | 0.0041 | yes |  |  |
| 4 | 6293659 | rs141233896 | C | G | missense | WFS1 | 0.0002 |  |  |  |
| 4 | 6296872 | rs142428158 | G | A | missense | WFS1 | 0.0002 | yes |  |  |
| 4 | 6302499 | rs369795224 | C | T | missense | WFS1 | 0.00002471 | yes |  |  |
| 4 | 6302816 | rs35031397 | C | G | missense | WFS1 | 0.0036 | yes |  |  |
| 4 | 6302843 | rs150894674 | G | A | missense | WFS1 | 0.0003 | yes | 600965 | DFNA6/14/38 |
| 4 | 6302889 | rs1801208 | G | A | missense | WFS1 | 0.057 | yes |  |  |
| 4 | 6302955 | rs377726402 | G | A | Nosense | WFS1 | 0.00000825 | yes |  |  |
| 4 | 6303011 | rs141254874 | G | C | missense | WFS1 | 8.249E-06 |  |  |  |
| 4 | 6303033 | rs28937892 | C | T | missense | WFS1 | 0.0000495 | yes |  |  |
| 4 | 6303080 | rs377544135 | C | G | missense | WFS1 | 0.00004951 | yes |  |  |
| 4 | 6303179 | rs150840308 | G | A | missense | WFS1 | 0.00009071 |  |  |  |
| 4 | 6303194 | rs199946797 | C | T | missense | WFS1 | 0.0005 | yes |  |  |
| 4 | 6303248 | rs1805069 | G | A | missense | WFS1 | 0.0089 | yes |  |  |
| 4 | 6303278 | rs143084511 | G | T | missense | WFS1 | 0 | yes |  |  |
| 4 | 6303281 | rs138968466 | C | T | missense | WFS1 | 0.00007419 |  |  |  |
| 4 | 6303306 | rs148544389 | C | T | missense | WFS1 | 0 |  |  |  |
| 4 | 6303361 | rs143064649 | G | A | Nosense | WFS1 | 0.00000824 | yes |  |  |
| 4 | 6303422 | rs140213376 | A | C | missense | WFS1 | 8.241E-06 |  |  |  |
| 4 | 6303516 | rs138258392 | C | T | missense | WFS1 | 0 |  |  |  |
| 4 | 6303534 | rs71530907 | C | T | missense | WFS1 | 0.007 | yes |  |  |
| 4 | 6303641 | rs71524377 | G | A | missense | WFS1 | 0.00004207 | yes |  |  |
| 4 | 6303644 | rs200099217 | C | T | missense | WFS1 | 0.0001 | yes |  |  |
| 4 | 6303660 | rs143280847 | A | G | missense | WFS1 | 0.00004235 |  |  |  |
| 4 | 6303680 | rs1805070 | A | G | missense | WFS1 | 0.0074 | yes |  |  |
| 4 | 6303776 | rs201239579 | G | T | Nosense | WFS1 | 8.691E-06 | yes |  |  |
| 4 | 6303869 | rs71526461 | T | C | missense | WFS1 | 0.0001 |  |  |  |
| 4 | 6303887 | rs376974936 | G | A | missense | WFS1 | 0.00005197 |  |  |  |
| 4 | 6303891 | rs369107336 | C | G | missense | WFS1 | 0.0000347 |  |  |  |
| 4 | 6304188 | rs147934586 | C | T | missense | WFS1 | 0.00001705 |  |  |  |
| 4 | 88533540 | rs36094464 | A | T | missense | DSPP | 0.0905 | yes |  |  |
| 4 | 88533843 | rs368559431 | G | A | missense | DSPP | 0.00001658 |  |  |  |
| 4 | 88534138 | rs200819405 | C | A | missense | DSPP | 0.00003313 |  |  |  |
| 4 | 88534326 | rs201942511 | G | A | missense | DSPP | 0.0004 |  |  |  |
| 4 | 88535112 | rs368812371 | G | T | missense | DSPP | 0.00002485 |  |  |  |
| 4 | 88536188 | rs201148490 | A | G | missense | DSPP | 0.0014 |  | 605594 | DFNA39 |
| 4 | 88536269 | rs371825362 | G | A | missense | DSPP | 0.0004 |  |  |  |
| 4 | 88536362 | rs111205174 | G | A | missense | DSPP | 0.505 |  |  |  |
| 4 | 88536650 | rs370270012 | G | T | missense | DSPP | 0.0001 |  |  |  |
| 4 | 88537715 | rs148827799 | G | T | missense | DSPP | 0.0003 |  |  |  |
| 5 | 140908057 | rs376328260 | A | C | missense | DIAPH1 | 0.00001656 |  | 124900 | DFNA1 |
| 5 | 145719411 | rs139312280 | C | A | missense | POU4F3 | 0.00005821 |  |  |  |
| 5 | 145719480 | rs372436251 | C | T | missense | POU4F3 | 0.00004173 |  | 602459 | DFNA15 |
| 5 | 145719481 | rs367737951 | C | T | missense | POU4F3 | 0.00001669 |  |  |  |
| 5 | 145719516 | rs368239745 | T | G | missense | POU4F3 | 0.00002509 |  |  |  |
| 6 | 33133557 | rs377656039 | G | C | missense | COL11A2 | 0 |  |  |  |
| 6 | 33137619 | rs142500487 | G | A | missense | COL11A2 | 0.00001648 |  |  |  |
| 6 | 33141825 | rs121912949 | G | A | missense | COL11A2 | 0.0001 | yes |  |  |
| 6 | 33142318 | rs376355040 | G | A | missense | COL11A2 | 8.946E-06 |  |  |  |
| 6 | 33144056 | rs141023125 | T | G | missense | COL11A2 | 0.0000113 |  | 601868 | DFNA13 |
| 6 | 33146726 | rs149697159 | G | C | missense | COL11A2 | 0.0000934 | yes |  |  |
| 6 | 33146747 | rs145499142 | G | A | missense | COL11A2 | 0.0011 | yes |  |  |
| 6 | 33147579 | rs144862714 | G | A | missense | COL11A2 | 0.0001 | yes |  |  |
| 6 | 33156764 | rs138305560 | G | C | missense | COL11A2 | 0.00004969 |  |  |  |
| 6 | 76527343 | rs371575926 | G | A | missense | MYO6 | 0.00003295 |  |  |  |
| 6 | 76550343 | rs150820400 | C | T | missense | MYO6 | 0.00004149 |  |  |  |
| 6 | 76572432 | rs369889326 | C | T | Nosense | MYO6 | 0.00000824 |  | 606346 | DFNA22 |
| 6 | 76599811 | rs370750657 | A | G | missense | MYO6 | 0.00001666 |  |  |  |
| 6 | 76621394 | rs141925339 | G | A | missense | MYO6 | 0.00001661 |  |  |  |
| 6 | 76624662 | rs367978681 | A | G | missense | MYO6 | 0.00005818 |  |  |  |
| 8 | 102643928 | rs200016612 | G | C | missense | GRHL2 | 0.0001 |  | 608641 | DFNA28 |
| 9 | 71845108 | rs142684074 | C | T | missense | TJP2 | 0.00002471 |  |  |  |
| 9 | 71855051 | rs143346845 | C | T | missense | TJP2 | 0.00001785 |  | 613558 | DFNA51 |
| 9 | 71863070 | rs28556975 | T | C | missense | TJP2 | 0.0029 |  |  |  |
| 9 | 75303654 | rs140437301 | G | A | missense | TMC1 | 0.0002 |  |  |  |
| 9 | 75366775 | rs199560971 | G | A | missense | TMC1 | 0.00001648 |  |  |  |
| 9 | 75387348 | rs375919123 | T | C | missense | TMC1 | 8.237E-06 |  |  |  |
| 9 | 75403306 | rs148443938 | T | G | missense | TMC1 | 0.00001648 |  | 606705 | DFNA36 |
| 9 | 75404123 | rs367924428 | G | A | missense | TMC1 | 0.00007425 | yes |  |  |
| 9 | 75404174 | rs151001642 | C | T | Nosense | TMC1 | 0.00001648 | yes |  |  |
| 9 | 75406910 | rs372710475 | C | T | missense | TMC1 | 0.00008237 | yes |  |  |
| 9 | 75435758 | rs368084452 | G | A | missense | TMC1 | 8.247E-06 | yes |  |  |
| 9 | 117803271 | rs2274750 | C | T | missense | TNC | 0.0516 | yes |  |  |
| 9 | 117808785 | rs2104772 | T | A | missense | TNC | 0.4385 | yes |  |  |
| 9 | 117819465 | rs200005353 | G | A | missense | TNC | 0.00006629 |  |  |  |
| 9 | 117822050 | rs148749117 | C | T | missense | TNC | 0.00006629 |  |  |  |
| 9 | 117825276 | rs373148389 | G | T | missense | TNC | 8.288E-06 |  |  |  |
| 9 | 117827085 | rs141417605 | C | T | missense | TNC | 8.237E-06 |  | 615629 | DFNA56 |
| 9 | 117827169 | rs369874534 | C | T | missense | TNC | 0.0000825 |  |  |  |
| 9 | 117840353 | rs142334930 | G | A | missense | TNC | 0.00008237 |  |  |  |
| 9 | 117848284 | rs371055558 | C | T | missense | TNC | 0.00002537 |  |  |  |
| 9 | 117848760 | rs141624690 | C | T | missense | TNC | 0.00002471 |  |  |  |
| 9 | 117849138 | rs141281085 | C | T | missense | TNC | 8.238E-06 |  |  |  |
| 9 | 117849280 | rs150493993 | C | T | missense | TNC | 0.00009069 |  |  |  |
| 9 | 117853183 | rs143586851 | C | T | missense | TNC | 0.0002 |  |  |  |
| 11 | 76853783 | rs1052030 | T | C | missense | MYO7A | 0.4348 | yes |  |  |
| 11 | 76853790 | rs371849195 | G | C | missense | MYO7A | 0.00005013 |  |  |  |
| 11 | 76867062 | rs370395532 | C | G | missense | MYO7A | 0.00001716 | yes |  |  |
| 11 | 76867967 | rs201539845 | G | A | missense | MYO7A | 0.00003318 | yes |  |  |
| 11 | 76868016 | rs370897466 | A | C | missense | MYO7A | 8.325E-06 |  |  |  |
| 11 | 76868392 | rs184866544 | A | G | missense | MYO7A | 0.0011 | yes |  |  |
| 11 | 76870496 | rs45629132 | G | A | missense | MYO7A | 0.0011 | yes |  |  |
| 11 | 76871254 | rs368716988 | A | G | missense | MYO7A | 0.0003 |  |  |  |
| 11 | 76873225 | rs200304238 | A | G | missense | MYO7A | 0.0002 |  |  |  |
| 11 | 76873944 | rs375350389 | C | G | missense | MYO7A | 8.283E-06 |  |  |  |
| 11 | 76885871 | rs111033201 | C | T | Nosense | MYO7A | 0.00003682 | yes |  |  |
| 11 | 76885947 | rs200057810 | C | T | missense | MYO7A | 0.00005899 |  | 601317 | DFNA11 |
| 11 | 76890889 | rs368341987 | G | A | missense | MYO7A | 0.0039 | yes |  |  |
| 11 | 76892489 | rs373089701 | C | T | missense | MYO7A | 0.00003807 |  |  |  |
| 11 | 76892561 | rs375668125 | G | A | missense | MYO7A | 8.569E-06 |  |  |  |
| 11 | 76892613 | rs199575418 | G | A | missense | MYO7A | 0.0002 |  |  |  |
| 11 | 76893620 | rs375050157 | T | A | missense | MYO7A | 0.00002515 | yes |  |  |
| 11 | 76901153 | rs111033178 | G | A | missense | MYO7A | 0.0004 | yes |  |  |
| 11 | 76903189 | rs376291076 | G | A | missense | MYO7A | 0.0001 | yes |  |  |
| 11 | 76910708 | rs41298747 | C | T | missense | MYO7A | 0.005 | yes |  |  |
| 11 | 76912636 | rs2276288 | A | T | missense | MYO7A | 0.544 | yes |  |  |
| 11 | 76914163 | rs111033287 | C | T | missense | MYO7A | 0.0021 | yes |  |  |
| 11 | 76915143 | rs201008835 | C | A | missense | MYO7A | 0.0002 |  |  |  |
| 11 | 76915183 | rs376674270 | G | A | missense | MYO7A | 0.00007701 |  |  |  |
| 11 | 76916599 | rs368657015 | T | C | missense | MYO7A | 0.00003079 | yes |  |  |
| 11 | 76924054 | rs367647666 | G | A | missense | MYO7A | 0.00007811 |  |  |  |
| 11 | 76925708 | rs200359303 | G | A | missense | MYO7A | 0.0003 |  |  |  |
| 11 | 120976653 | rs376541939 | G | A | missense | TECTA | 8.236E-06 |  |  |  |
| 11 | 120979969 | rs145898158 | C | T | missense | TECTA | 0.00003301 | yes |  |  |
| 11 | 120998925 | rs371892292 | C | T | missense | TECTA | 0.00001658 |  |  |  |
| 11 | 120999013 | rs374863954 | A | T | missense | TECTA | 0.00003398 |  |  |  |
| 11 | 121000423 | rs111759871 | C | T | missense | TECTA | 0.0002 | yes |  |  |
| 11 | 121000636 | rs146175803 | A | G | missense | TECTA | 0.0004 | yes |  |  |
| 11 | 121000716 | rs143998942 | G | A | missense | TECTA | 0.00007547 |  |  |  |
| 11 | 121000878 | rs374229006 | G | C | missense | TECTA | 0.00002517 |  | 601543 | DFNA8/12 |
| 11 | 121008565 | rs369690173 | T | G | missense | TECTA | 8.324E-06 |  |  |  |
| 11 | 121008594 | rs147890616 | G | C | missense | TECTA | 0.0001 | yes |  |  |
| 11 | 121008681 | rs373132598 | G | A | missense | TECTA | 0.00003336 |  |  |  |
| 11 | 121016729 | rs373655409 | G | A | missense | TECTA | 0.00004191 |  |  |  |
| 11 | 121023709 | rs375984509 | G | C | missense | TECTA | 0 |  |  |  |
| 11 | 121028738 | rs374996667 | C | A | missense | TECTA | 0 |  |  |  |
| 11 | 121038716 | rs367589125 | T | A | missense | TECTA | 0.00002473 |  |  |  |
| 11 | 121038773 | rs140236996 | C | T | missense | TECTA | 8.237E-06 | yes |  |  |
| 12 | 57422595 | rs370014993 | T | C | missense | MYO1A | 0.00005766 |  |  |  |
| 12 | 57424918 | rs113470661 | G | A | missense | MYO1A | 0.0047 | yes |  |  |
| 12 | 57430769 | rs138855953 | C | T | missense | MYO1A | 0.00002471 |  |  |  |
| 12 | 57430791 | rs373952237 | G | A | missense | MYO1A | 0.00002471 |  |  |  |
| 12 | 57431366 | rs148808080 | C | T | missense | MYO1A | 0.0005 | yes |  |  |
| 12 | 57431698 | rs144320005 | C | T | Nosense | MYO1A | 0.00006627 |  |  |  |
| 12 | 57431785 | rs368223948 | C | T | missense | MYO1A | 0 |  | 608652 | DFNA48 |
| 12 | 57432715 | rs367561406 | T | C | missense | MYO1A | 0.00002475 |  |  |  |
| 12 | 57435225 | rs61753849 | C | A | missense | MYO1A | 0.00009884 | yes |  |  |
| 12 | 57437119 | rs55679042 | C | T | missense | MYO1A | 0.0055 | yes |  |  |
| 12 | 57437952 | rs137975387 | G | C | missense | MYO1A | 0.0004 |  |  |  |
| 12 | 57440417 | rs146269737 | G | A | Nosense | MYO1A | 0.00001648 |  |  |  |
| 12 | 57441459 | rs121909305 | G | A | Nosense | MYO1A | 0.0032 | yes |  |  |
| 12 | 100806604 | rs373873276 | G | A | missense | SLC17A8 | 0.00001647 |  | 605583 | DFNA25 |
| 12 | 100813782 | rs372802080 | T | G | missense | SLC17A8 | 0 |  |  |  |
| 12 | 133197122 | rs149982621 | T | C | missense | P2RX2 | 0.00006615 |  |  |  |
| 12 | 133197906 | rs147592928 | G | A | missense | P2RX2 | 0.00002474 |  | 608224 | DFNA41 |
| 12 | 133198367 | rs140087499 | C | T | missense | P2RX2 | 0.00009463 |  |  |  |
| 13 | 20763045 | rs370868313 | C | T | missense | GJB2 | 0 |  |  |  |
| 13 | 20763051 | rs111033194 | T | G | missense | GJB2 | 0.00009471 | yes |  |  |
| 13 | 20763104 | rs111033294 | T | C | missense | GJB2 | 0.00008391 | yes |  |  |
| 13 | 20763222 | rs111033360 | C | T | missense | GJB2 | 0.00003304 | yes |  |  |
| 13 | 20763246 | rs373684994 | C | T | missense | GJB2 | 0.0001 | yes |  |  |
| 13 | 20763269 | rs370044106 | A | G | missense | GJB2 | 0.00000826 | yes |  |  |
| 13 | 20763294 | rs80338948 | G | A | missense | GJB2 | 0.0002 | yes |  |  |
| 13 | 20763341 | rs111033196 | C | T | missense | GJB2 | 0.0154 | yes | 601544 | DFNA3A |
| 13 | 20763366 | rs150529554 | C | T | missense | GJB2 | 0.00009913 | yes |  |  |
| 13 | 20763395 | rs374572413 | C | T | missense | GJB2 | 0.00002477 | yes |  |  |
| 13 | 20763452 | rs80338945 | A | G | missense | GJB2 | 0.0009 | yes |  |  |
| 13 | 20763472 | rs111033218 | G | C | missense | GJB2 | 0.0018 | yes |  |  |
| 13 | 20763483 | rs199883710 | G | A | Nosense | GJB2 | 8.239E-06 | yes |  |  |
| 13 | 20763534 | rs370696868 | C | T | missense | GJB2 | 0.00001648 | yes |  |  |
| 13 | 20763602 | rs111033296 | G | T | missense | GJB2 | 8.244E-06 | yes |  |  |
| 13 | 20763627 | rs371024165 | G | A | missense | GJB2 | 0.0000412 | yes |  |  |
| 13 | 20763642 | rs2274084 | C | T | missense | GJB2 | 0.0454 | yes |  |  |
| 13 | 20797001 | rs146231737 | C | T | missense | GJB6 | 0.00005767 |  | 612643 | DFNA3B |
| 14 | 31344166 | rs200935305 | G | A | missense | COCH | 0.0002 |  | 601369 | DFNA9 |
| 14 | 31355287 | rs367884240 | C | T | missense | COCH | 0.0000412 |  |  |  |
| 14 | 61113177 | rs144481204 | C | A | missense | SIX1 | 0.00006589 | yes | 605192 | DFNA23 |
| 16 | 2546346 | rs371245371 | C | T | missense | TBC1D24 | 0.00002508 |  |  |  |
| 16 | 2547101 | rs370233833 | G | A | missense | TBC1D24 | 8.516E-06 |  | 613577 | DFNA65 |
| 16 | 2550904 | rs372995761 | A | G | missense | TBC1D24 | 8.397E-06 |  |  |  |
| 19 | 50720992 | rs138001307 | G | A | missense | MYH14 | 0.0002 |  |  |  |
| 19 | 50728854 | rs371766484 | C | T | missense | MYH14 | 0.00001999 |  |  |  |
| 19 | 50747534 | rs119103280 | G | T | missense | MYH14 | 0.0029 | yes | 600652 | DFNA4 |
| 19 | 50771512 | rs113993956 | G | A | missense | MYH14 | 0.0004 | yes |  |  |
| 19 | 50792886 | rs368076336 | G | A | missense | MYH14 | 0 |  |  |  |
| 19 | 50794165 | rs375795690 | C | T | missense | MYH14 | 0.00008387 |  |  |  |
| 22 | 36678809 | rs142565774 | C | T | missense | MYH9 | 0.00007722 |  |  |  |
| 22 | 36682852 | rs375515914 | C | T | missense | MYH9 | 0.00002473 |  |  |  |
| 22 | 36682873 | rs142094977 | A | G | missense | MYH9 | 0.0013 | yes |  |  |
| 22 | 36684873 | rs373393111 | C | T | missense | MYH9 | 0.00001648 | yes | 160775 | DFNA17 |
| 22 | 36688178 | rs76368635 | G | A | missense | MYH9 | 0.001 | yes |  |  |
| 22 | 36691696 | rs200901330 | A | G | missense | MYH9 | 0.0003 | yes |  |  |
| 22 | 36692971 | rs147911658 | T | A | missense | MYH9 | 8.291E-06 |  |  |  |
| 22 | 36710207 | rs375899392 | C | T | missense | MYH9 | 0.00001648 |  |  |  |

**Table S2** Two hundred randomly selected SNV located in genes causing Centro Nuclear Myopathy.

| CHR. | POSITION | Rs | REF | ALT | FUNTION | GEN | MAF | HMDB | PHENOTYPE MIM NUMBER | DISEASE |
| --- | --- | --- | --- | --- | --- | --- | --- | --- | --- | --- |
| 2 | 127806106 | rs375004668 | G | A | missense | BIN1 | 0.0001 |  |  |  |
| 2 | 127806143 | rs147655157 | G | A | missense | BIN1 | 0.0002 |  |  |  |
| 2 | 127806161 | rs121909275 | T | A | nosense | BIN1 | 0.0001 | yes |  |  |
| 2 | 127806176 | rs368983991 | C | T | missense | BIN1 | 0.0001 |  |  |  |
| 2 | 127808046 | rs138047593 | T | C | missense | BIN1 | 0.003 |  |  |  |
| 2 | 127808076 | rs112318500 | G | A | missense | BIN1 | 0.034 |  |  |  |
| 2 | 127808410 | rs148422103 | G | A | missense | BIN1 | 0.0008 |  |  |  |
| 2 | 127808434 | rs371571307 | C | T | missense | BIN1 | 0.0001 |  |  |  |
| 2 | 127808458 | rs368238742 | A | G | missense | BIN1 | 0.0001 |  |  |  |
| 2 | 127808470 | rs144459969 | C | T | missense | BIN1 | 0.0002 |  |  |  |
| 2 | 127808746 | rs372650268 | G | C | missense | BIN1 | 0.0001 |  | 255200 | CNM2 |
| 2 | 127808749 | rs140410496 | G | A | missense | BIN1 | 0.0001 |  |  |  |
| 2 | 127809920 | rs200124094 | C | T | missense | BIN1 | 0.0002 |  |  |  |
| 2 | 127811539 | rs375322787 | T | C | missense | BIN1 | 0.0001 |  |  |  |
| 2 | 127811566 | rs368616652 | G | A | missense | BIN1 | 0.0001 |  |  |  |
| 2 | 127811582 | rs200887814 | C | T | missense | BIN1 | 0.0004 |  |  |  |
| 2 | 127815174 | rs76037557 | G | A | missense | BIN1 | 0.0002 |  |  |  |
| 2 | 127816664 | rs374565677 | C | T | missense | BIN1 | 0.0003 |  |  |  |
| 2 | 127818175 | rs367585396 | T | A | missense | BIN1 | 0.0001 |  |  |  |
| 2 | 127818193 | rs117721706 | C | T | missense | BIN1 | 0.0046 |  |  |  |
| 2 | 127818194 | rs144391901 | G | A | missense | BIN1 | 0.0002 |  |  |  |
| 2 | 127818197 | rs148473945 | A | G | missense | BIN1 | 0.0005 |  |  |  |
| 2 | 127819743 | rs372072916 | C | T | missense | BIN1 | 0.0001 |  |  |  |
| 2 | 127821184 | rs375697182 | G | A | missense | BIN1 | 0.0001 |  |  |  |
| 2 | 127821206 | rs146573197 | C | T | missense | BIN1 | 0.0004 |  |  |  |
| 2 | 127821511 | rs143820618 | G | T | missense | BIN1 | 0.0004 |  |  |  |
| 2 | 127826543 | rs371755655 | G | T | missense | BIN1 | 0.0001 |  |  |  |
| 2 | 127826558 | rs267606681 | C | T | missense | BIN1 | 0.000001 | yes |  |  |
| 2 | 127826568 | rs121909274 | C | T | missense | BIN1 | 0.000001 | yes |  |  |
| 2 | 127834212 | rs369549551 | T | A | missense | BIN1 | 0.0001 |  |  |  |
| 2 | 127834262 | rs121909273 | C | A | missense | BIN1 | 0.000001 | yes |  |  |
| 2 | 127864463 | rs142657993 | C | G | missense | BIN1 | 0.0001 |  |  |  |
| 3 | 9695311 | rs377332766 | C | G | missense | MTMR14 | 0.0001 |  |  |  |
| 3 | 9695332 | rs375944156 | G | T | nosense | MTMR14 | 0.0001 |  |  |  |
| 3 | 9704013 | rs368196455 | G | A | missense | MTMR14 | 0.0001 |  |  |  |
| 3 | 9704024 | rs372268047 | C | T | missense | MTMR14 | 0.0001 |  |  |  |
| 3 | 9711119 | rs373227805 | G | C | missense | MTMR14 | 0.0001 |  |  |  |
| 3 | 9711141 | rs189614064 | T | A | missense | MTMR14 | 0.001 |  |  |  |
| 3 | 9712833 | rs374591212 | A | G | missense | MTMR14 | 0.0001 |  |  |  |
| 3 | 9714412 | rs200924533 | G | A | missense | MTMR14 | 0.0001 |  |  |  |
| 3 | 9714418 | rs201904466 | A | G | missense | MTMR14 | 0.0002 |  |  |  |
| 3 | 9719029 | rs142525507 | T | A | missense | MTMR14 | 0.0008 |  |  |  |
| 3 | 9719057 | rs374251047 | T | C | missense | MTMR14 | 0.0001 |  | 160150 | CNM1 |
| 3 | 9719695 | rs372538745 | C | T | missense | MTMR14 | 0.0001 |  |  |  |
| 3 | 9726277 | rs375469777 | G | A | missense | MTMR14 | 0.0001 |  |  |  |
| 3 | 9726311 | rs121434509 | G | A | missense | MTMR14 | 0.000001 | yes |  |  |
| 3 | 9726588 | rs183134138 | C | T | missense | MTMR14 | 0.0014 |  |  |  |
| 3 | 9726918 | rs115607360 | G | A | missense | MTMR14 | 0.0006 |  |  |  |
| 3 | 9729559 | rs372498357 | G | A | missense | MTMR14 | 0.0002 |  |  |  |
| 3 | 9730400 | rs369183361 | G | A | missense | MTMR14 | 0.0001 |  |  |  |
| 3 | 9730639 | rs376068526 | C | T | missense | MTMR14 | 0.0002 |  |  |  |
| 3 | 9730643 | rs377445755 | G | A | missense | MTMR14 | 0.0001 |  |  |  |
| 3 | 9730675 | rs370380809 | C | G | missense | MTMR14 | 0.0001 |  |  |  |
| 3 | 9730678 | rs374725262 | G | A | missense | MTMR14 | 0.0001 |  |  |  |
| 3 | 9730693 | rs371569636 | C | T | missense | MTMR14 | 0.0001 |  |  |  |
| 3 | 9730709 | rs375373181 | G | A | missense | MTMR14 | 0.0001 |  |  |  |
| 3 | 9730718 | rs121434510 | G | A | missense | MTMR14 | 0.000001 | yes |  |  |
| 3 | 9730758 | rs371363549 | G | C | missense | MTMR14 | 0.0001 |  |  |  |
| 3 | 9731692 | rs201206576 | G | A | missense | MTMR14 | 0.0002 |  |  |  |
| 3 | 9739406 | rs371144090 | A | G | missense | MTMR14 | 0.0001 |  |  |  |
| 3 | 9739439 | rs201626220 | A | G | missense | MTMR14 | 0.0006 |  |  |  |
| 3 | 9739479 | rs376164405 | G | C | missense | MTMR14 | 0.0001 |  |  |  |
| 3 | 9739498 | rs370895091 | A | G | missense | MTMR14 | 0.0001 |  |  |  |
| 3 | 9739526 | rs200360764 | C | T | missense | MTMR14 | 0.0001 |  |  |  |
| 3 | 9743502 | rs368605936 | C | T | missense | MTMR14 | 0.0001 |  |  |  |
| 3 | 9743503 | rs370811714 | G | A | missense | MTMR14 | 0.0001 |  |  |  |
| 3 | 9743528 | rs374180282 | C | G | missense | MTMR14 | 0.0001 |  |  |  |
| 3 | 9743616 | rs375966737 | C | T | missense | MTMR14 | 0.0001 |  |  |  |
| 3 | 9743632 | rs202121982 | G | A | missense | MTMR14 | 0.0005 |  |  |  |
| 12 | 81101577 | rs147184101 | G | A | missense | MYF6 | 0.0002 |  |  |  |
| 12 | 81101682 | rs190471225 | G | A | missense | MYF6 | 0.0004 |  |  |  |
| 12 | 81101767 | rs138296448 | C | A | missense | MYF6 | 0.0004 | yes |  |  |
| 12 | 81101770 | rs372392737 | C | T | missense | MYF6 | 0.0001 |  | 614408 | CNM3 |
| 12 | 81101786 | rs377370090 | A | T | missense | MYF6 | 0.0001 |  |  |  |
| 12 | 81101832 | rs28928909 | G | T | missense | MYF6 | 0.0006 | yes |  |  |
| 12 | 81101845 | rs200372502 | T | C | missense | MYF6 | 0.0001 |  |  |  |
| 12 | 81101886 | rs370270818 | A | G | missense | MYF6 | 0.0002 |  |  |  |
| 12 | 81101976 | rs368477055 | C | G | missense | MYF6 | 0.0001 |  |  |  |
| 12 | 81102342 | rs143677057 | T | A | missense | MYF6 | 0.0004 |  |  |  |
| 12 | 81102358 | rs146824657 | A | C | missense | MYF6 | 0.0002 |  |  |  |
| 12 | 81102363 | rs375170162 | T | C | missense | MYF6 | 0.0001 |  |  |  |
| 12 | 81102373 | rs143786238 | T | C | missense | MYF6 | 0.0001 |  |  |  |
| 12 | 81102385 | rs375228457 | C | A | missense | MYF6 | 0.0001 |  |  |  |
| 19 | 10870442 | rs144250390 | G | A | missense | DNM2 | 0.0022 |  |  |  |
| 19 | 10883157 | rs148790687 | C | T | missense | DNM2 | 0.0002 |  |  |  |
| 19 | 10883206 | rs369347296 | A | G | missense | DNM2 | 0.0001 |  |  |  |
| 19 | 10883235 | rs375151459 | G | A | missense | DNM2 | 0.0002 |  |  |  |
| 19 | 10886432 | rs370086632 | G | A | missense | DNM2 | 0.0001 |  |  |  |
| 19 | 10887847 | rs145478270 | G | A | missense | DNM2 | 0.0004 |  |  |  |
| 19 | 10893725 | rs145607989 | C | G | missense | DNM2 | 0.0002 |  |  |  |
| 19 | 10893758 | rs138128705 | C | T | missense | DNM2 | 0.0001 |  |  |  |
| 19 | 10893786 | rs202155679 | C | T | missense | DNM2 | 0.0006 |  |  |  |
| 19 | 10897265 | rs140963588 | C | T | missense | DNM2 | 0.0001 |  |  |  |
| 19 | 10897334 | rs148105340 | A | G | missense | DNM2 | 0.0001 |  |  |  |
| 19 | 10904505 | rs121909092 | G | A | missense | DNM2 | 0.0001 | yes | 160150 | CNM1 |
| 19 | 10904508 | rs121909090 | C | T | missense | DNM2 | 0.01 | yes |  |  |
| 19 | 10904509 | rs121909089 | G | A | missense | DNM2 | 0.03 | yes |  |  |
| 19 | 10906825 | rs140208362 | G | A | missense | DNM2 | 0.0001 |  |  |  |
| 19 | 10908100 | rs199927590 | A | G | missense | DNM2 | 0.0001 |  |  |  |
| 19 | 10908108 | rs148633841 | A | G | missense | DNM2 | 0.0001 |  |  |  |
| 19 | 10908190 | rs371514802 | A | G | missense | DNM2 | 0.0001 |  |  |  |
| 19 | 10909177 | rs372593558 | C | T | missense | DNM2 | 0.0001 |  |  |  |
| 19 | 10909184 | rs140043676 | G | A | missense | DNM2 | 0.0003 |  |  |  |
| 19 | 10909199 | rs375653221 | G | A | missense | DNM2 | 0.0001 |  |  |  |
| 19 | 10909204 | rs143992936 | G | A | missense | DNM2 | 0.0001 |  |  |  |
| 19 | 10909219 | rs121909091 | C | T | missense | DNM2 | 0.000001 | yes |  |  |
| 19 | 10916639 | rs141132980 | A | G | missense | DNM2 | 0.0001 |  |  |  |
| 19 | 10922976 | rs368752035 | A | G | missense | DNM2 | 0.0001 |  |  |  |
| 19 | 10922991 | rs121909093 | G | T | missense | DNM2 | 0.000001 | yes |  |  |
| 19 | 10923027 | rs144763522 | T | A | missense | DNM2 | 0.0001 |  |  |  |
| 19 | 10930668 | rs121909088 | A | G | missense | DNM2 | 0.11 | yes |  |  |
| 19 | 10930693 | rs121909094 | T | A | missense | DNM2 | 0.14 | yes |  |  |
| 19 | 10934538 | rs121909095 | C | T | missense | DNM2 | 0.02 | yes |  |  |
| 19 | 10934538 | rs121909096 | C | G | missense | DNM2 | 0.000001 | yes |  |  |
| 19 | 10939829 | rs370459176 | T | C | missense | DNM2 | 0.0002 |  |  |  |
| 19 | 10939902 | rs375350902 | C | G | missense | DNM2 | 0.0001 |  |  |  |
| 19 | 10939910 | rs368325934 | G | A | missense | DNM2 | 0.0001 |  |  |  |
| 19 | 10939922 | rs375820696 | C | T | missense | DNM2 | 0.0002 |  |  |  |
| 19 | 10940816 | rs374864354 | C | T | missense | DNM2 | 0.0002 |  |  |  |
| 19 | 10940928 | rs138527500 | C | T | missense | DNM2 | 0.0001 |  |  |  |
| 19 | 10941659 | rs139930306 | G | C | missense | DNM2 | 0.0001 |  |  |  |
| 19 | 10941677 | rs149825590 | G | A | missense | DNM2 | 0.0003 |  |  |  |
| 19 | 10941695 | rs369312570 | G | A | missense | DNM2 | 0.0001 |  |  |  |
| 19 | 10943688 | rs139213045 | G | A | missense | DNM2 | 0.0005 |  |  |  |
| 19 | 10943750 | rs146430642 | G | A | missense | DNM2 | 0.0004 |  |  |  |
| 19 | 10943768 | rs373157246 | C | T | nosense | DNM2 | 0.0001 |  |  |  |
| 19 | 10943807 | rs376139740 | C | T | missense | DNM2 | 0.0002 |  |  |  |
| 19 | 10943828 | rs370918190 | C | T | missense | DNM2 | 0.0001 |  |  |  |
| 19 | 10943855 | rs151223408 | C | T | missense | DNM2 | 0.0002 |  |  |  |
| 19 | 10943856 | rs373835440 | G | A | missense | DNM2 | 0.0001 |  |  |  |
| 19 | 10943882 | rs372270914 | A | G | missense | DNM2 | 0.0001 |  |  |  |
| 19 | 38939430 | rs140037232 | C | T | missense | RYR1 | 0.0002 | yes |  |  |
| 19 | 38945887 | rs147723844 | A | G | missense | RYR1 | 0.001 | yes |  |  |
| 19 | 38946103 | rs111888148 | G | A | missense | RYR1 | 0.0002 | yes |  |  |
| 19 | 38946112 | rs144336148 | G | A | missense | RYR1 | 0.0005 | yes |  |  |
| 19 | 38948186 | rs193922772 | G | A | missense | RYR1 | 0.0001 | yes |  |  |
| 19 | 38948830 | rs144845360 | G | A | missense | RYR1 | 0.0002 |  |  |  |
| 19 | 38951020 | rs200069592 | G | A | missense | RYR1 | 0.0002 |  |  |  |
| 19 | 38951140 | rs372652716 | G | A | missense | RYR1 | 0.0001 |  |  |  |
| 19 | 38951205 | rs375669412 | G | A | missense | RYR1 | 0.0006 |  |  |  |
| 19 | 38954087 | rs138020885 | C | T | missense | RYR1 | 0.0002 |  |  |  |
| 19 | 38954405 | rs374492243 | A | G | missense | RYR1 | 0.0001 |  |  |  |
| 19 | 38954473 | rs144935444 | G | A | missense | RYR1 | 0.0001 |  |  |  |
| 19 | 38955289 | rs148623597 | G | A | missense | RYR1 | 0.0012 | yes |  |  |
| 19 | 38955362 | rs201827275 | C | T | missense | RYR1 | 0.0001 |  |  |  |
| 19 | 38956847 | rs367860207 | C | T | missense | RYR1 | 0.0001 |  |  |  |
| 19 | 38956858 | rs138209392 | G | A | missense | RYR1 | 0.0001 |  | 180901 | CNM |
| 19 | 38956954 | rs374477216 | C | T | missense | RYR1 | 0.0001 |  |  |  |
| 19 | 38956988 | rs374776563 | G | A | missense | RYR1 | 0.0002 |  |  |  |
| 19 | 38958382 | rs149096607 | C | T | missense | RYR1 | 0.0002 |  |  |  |
| 19 | 38959666 | rs201174268 | G | A | missense | RYR1 | 0.0002 |  |  |  |
| 19 | 38964116 | rs377185497 | C | T | missense | RYR1 | 0.0001 |  |  |  |
| 19 | 38964275 | rs34694816 | A | G | missense | RYR1 | 0.054 |  |  |  |
| 19 | 38966001 | rs141678782 | C | G | missense | RYR1 | 0.0001 |  |  |  |
| 19 | 38966014 | rs187496208 | C | T | missense | RYR1 | 0.0002 |  |  |  |
| 19 | 38966056 | rs150499158 | G | A | missense | RYR1 | 0.0001 |  |  |  |
| 19 | 38968395 | rs370851779 | G | A | missense | RYR1 | 0.0001 |  |  |  |
| 19 | 38976235 | rs368726019 | T | C | missense | RYR1 | 0.0001 |  |  |  |
| 19 | 38976331 | rs146504767 | G | A | missense | RYR1 | 0.0004 |  |  |  |
| 19 | 38976478 | rs193922781 | C | T | missense | RYR1 | 0.0001 | yes |  |  |
| 19 | 38976529 | rs377476955 | C | T | missense | RYR1 | 0.0001 |  |  |  |
| 19 | 38976636 | rs372958050 | T | C | missense | RYR1 | 0.0001 |  |  |  |
| 19 | 38976655 | rs34934920 | C | T | missense | RYR1 | 0.017 |  |  |  |
| 19 | 38976783 | rs147603571 | G | A | missense | RYR1 | 0.0001 |  |  |  |
| 19 | 38980791 | rs145801146 | C | T | missense | RYR1 | 0.0001 |  |  |  |
| 19 | 38985195 | rs143398211 | G | A | missense | RYR1 | 0.0002 | yes |  |  |
| 19 | 38986946 | rs193922795 | G | A | missense | RYR1 | 0.0001 | yes |  |  |
| 19 | 38989817 | rs34390345 | A | G | missense | RYR1 | 0.0002 | yes |  |  |
| 19 | 38989881 | rs147213895 | A | G | missense | RYR1 | 0.0008 | yes |  |  |
| 19 | 38989882 | rs202061237 | C | T | missense | RYR1 | 0.0004 | yes |  |  |
| 19 | 38990295 | rs193922802 | G | A | missense | RYR1 | 0.0001 | yes |  |  |
| 19 | 38990311 | rs144526634 | G | A | missense | RYR1 | 0.0002 | yes |  |  |
| 19 | 38990346 | rs146306934 | G | A | missense | RYR1 | 0.0002 | yes |  |  |
| 19 | 38990594 | rs193922808 | G | T | missense | RYR1 | 0.0001 | yes |  |  |
| 19 | 38991258 | rs375148516 | G | A | missense | RYR1 | 0.0001 |  |  |  |
| 19 | 38993605 | rs371447916 | C | T | missense | RYR1 | 0.0001 |  |  |  |
| 19 | 38995510 | rs2915951 | T | C | missense | RYR1 | 0.32 | yes |  |  |
| 19 | 38995998 | rs35180584 | C | G | missense | RYR1 | 0.009 | yes |  |  |
| 19 | 38996982 | rs138647599 | G | A | missense | RYR1 | 0.0001 |  |  |  |
| 19 | 39002892 | rs375292503 | A | G | missense | RYR1 | 0.0001 |  |  |  |
| 19 | 39003006 | rs61739911 | C | T | missense | RYR1 | 0.001 | yes |  |  |
| 19 | 39006812 | rs377541724 | A | G | missense | RYR1 | 0.0001 |  |  |  |
| 19 | 39009877 | rs118204421 | C | T | missense | RYR1 | 0.0002 | yes |  |  |
| 19 | 39010075 | rs148892609 | C | T | missense | RYR1 | 0.0001 |  |  |  |
| 19 | 39016132 | rs143987857 | G | A | missense | RYR1 | 0.0006 | yes |  |  |
| 19 | 39018342 | rs148130880 | G | A | missense | RYR1 | 0.0001 |  |  |  |
| 19 | 39026638 | rs140616359 | G | A | missense | RYR1 | 0.0002 | yes |  |  |
| 19 | 39034191 | rs147136339 | A | G | missense | RYR1 | 0.002 | yes |  |  |
| 19 | 39038899 | rs144685735 | C | T | missense | RYR1 | 0.0002 | yes |  |  |
| 19 | 39055615 | rs370527763 | G | A | missense | RYR1 | 0.0001 |  |  |  |
| 19 | 39057615 | rs73933023 | C | T | missense | RYR1 | 0.011 | yes |  |  |
| 19 | 39061260 | rs118192130 | G | A | missense | RYR1 | 0.0002 | yes |  |  |
| 19 | 39062672 | rs143520367 | C | T | missense | RYR1 | 0.0001 | yes |  |  |
| 19 | 39070706 | rs143988412 | A | G | missense | RYR1 | 0.0001 | yes |  |  |
| 19 | 39070708 | rs200442804 | C | T | missense | RYR1 | 0.0012 | yes |  |  |
| 19 | 39070725 | rs148540135 | C | T | missense | RYR1 | 0.0001 | yes |  |  |
| 19 | 39070731 | rs193922875 | G | A | missense | RYR1 | 0.0001 | yes |  |  |
| 19 | 39071022 | rs193922879 | G | A | missense | RYR1 | 0.0001 | yes |  |  |
| 19 | 39075653 | rs118192153 | C | T | missense | RYR1 | 0.0001 | yes |  |  |
| 19 | 39076780 | rs146876145 | C | T | missense | RYR1 | 0.0002 | yes |  |  |

**Table S3** Logit regression model to predict pathogenic variants is based on models consisting of single or multiple prediction tools for the top 10, 20 and 50 ranked variants for each tool, respectively. ROC curves and areas under the curve (AUC) values were used to compare the accuracy of each strategy. All p-value corrected by Bonferroni’s method are shown in parenthesis.

| **AD-SNHL** | **Pedigree** | **Area under curve** | **Top 10 AUC (p-value)** | **Top 20 AUC (p-value)** | **Top 50 AUC (p-value)** |
| --- | --- | --- | --- | --- | --- |
|  | 1 - F | **Combining 5 Systems** | 69% | 71% | 68% |
|  |  | **PAVAR** | 58% (6x10^-04^) | 59% (7x10^-05^) | 53% (4x10^-05^) |
|  |  | **Exomiser v2** | 51% (5x10^-05^) | 58% (4x10^-05^) | 58% (2x10^-04^) |
|  |  | **VAAST- Phevor** | 59% (6x10^-04^) | 59% (1x10^-03^) | 58% (5x10^-04^) |
|  |  | **CADD** | 55% (8x10^-05^) | 57% (1x10^-05^) | 50% (1x10^-05^) |
|  |  | **FATHMM** | 57% (2x10^-04^) | 51% (1x10^-06^) | 53% (9x10^-05^) |
|  | 1 - TF | **Combining 5 Systems** | 68% | 69% | 69% |
|  |  | **PAVAR** | 59% (2x10^-03^) | 60% (1x10^-03^) | 53% (2x10^-06^) |
|  |  | **Exomiser v2** | 51% (1x10^-04^) | 58% (2x10^-04^) | 57% (5x10^-04^) |
|  |  | **VAAST- Phevor** | 58% (1x10^-03^) | 59% (1x10^-03^) | 58% (6x10^-05^) |
|  |  | **CADD** | 52% (1x10^-04^) | 53% (1x10^-05^) | 50% (1x10^-06^) |
|  |  | **FATHMM** | 58% (1x10^-03^) | 51% (1x10^-05^) | 53% (6x10^-06^) |
|  | 1 - T | **Combining 5 Systems** | 66% | 70% | 65% |
|  |  | **PAVAR** | 55% (1x10^-03^) | 55% (1x10^-05^) | 53% (5x10^-04^) |
|  |  | **Exomiser v2** | 57% (4x10^-03^) | 59% (3x10^-03^) | 54% (4x10^-04^) |
|  |  | **VAAST- Phevor** | 58% (4x10^-03^) | 59% (1x10^-04^) | 59% (0.02) |
|  |  | **CADD** | 54% (7x10^-04^) | 54% (7x10^-06^) | 50% (2x10^-04^) |
|  |  | **FATHMM** | 55% (1x10^-03^) | 51% (2x10^-06^) | 51% (1x10^-04^) |
|  | 3 - F | **Combining 5 Systems** | 68% | 70% | 67% |
|  |  | **PAVAR** | 54% (1x10^-03^) | 52% (1x10^-04^) | 50% (8x10^-06^) |
|  |  | **Exomiser v2** | 59% (1x10^-03^) | 54% (2x10^-04^) | 54% (2x10^-03^) |
|  |  | **VAAST- Phevor** | 60% (1x10^-03^) | 59% (0.01) | 59% (2x10^-04^) |
|  |  | **CADD** | 52% (1x10^-04^) | 50% (7x10^-05^) | 50% (8x10^-06^) |
|  |  | **FATHMM** | 52% (1x10^-04^) | 53% (3x10^-04^) | 53% (4x10^-05^) |
|  | 3 - TF | **Combining 5 Systems** | 67% | 71% | 66% |
|  |  | **PAVAR** | 60% (0.02) | 54% (4x10^-06^) | 52% (2x10^-04^) |
|  |  | **Exomiser v2** | 54% (1x10^-03^) | 59% (2x10^-03^) | 54% (2x10^-03^) |
|  |  | **VAAST- Phevor** | 58% (8x10^-03^) | 62% (5x10^-04^) | 60% (0.03) |
|  |  | **CADD** | 55% (3x10^-03^) | 53% (1x10^-06^) | 50% (1x10^-04^) |
|  |  | **FATHMM** | 53% (1x10^-03^) | 51% (1x10^-06^) | 51% (1x10^-04^) |
|  | 3 - T | **Combining 5 Systems** | 73% | 66% | 66% |
|  |  | **PAVAR** | 54% (7x10^-07^) | 52% (1x10^-04^) | 50% (1x10^-04^) |
|  |  | **Exomiser v2** | 59% (4x10^-04^) | 54% (1x10^-03^) | 54% (1x10^-03^) |
|  |  | **VAAST- Phevor** | 61% (2x10^-05^) | 60% (7x10^-03^) | 60% (7x10^-03^) |
|  |  | **CADD** | 54% (4x10^-07^) | 50% (9x10^-05^) | 50% (9x10^-05^) |
|  |  | **FATHMM** | 50% (2x10^-07^) | 50% (6x10^-05^) | 50% (6x10^-05^) |
|  | 5 - F | **Combining 5 Systems** | 67% | 66% | 68% |
|  |  | **PAVAR** | 54% (5x10^-04^) | 53% (3x10^-03^) | 52% (8x10^-06^) |
|  |  | **Exomiser v2** | 58% (25x10^-03^) | 54% (2x10^-03^) | 57% (1x10^-03^) |
|  |  | **VAAST- Phevor** | 59% (3x10^-03^) | 58% (0.06) | 58% (1x10^-04^) |
|  |  | **CADD** | 54% (2x10^-04^) | 50% (1x10^-03^) | 50% (4x10^-06^) |
|  |  | **FATHMM** | 52% (1x10^-04^) | 53% (7x10^-03^) | 53% (2x10^-05^) |
|  | 5 - TF | **Combining 5 Systems** | 72% | 70% | 69% |
|  |  | **PAVAR** | 59% (1x10^-05^) | 60% (2x10^-04^) | 53% (1x10^-06^) |
|  |  | **Exomiser v2** | 58% (5x10^-06^) | 58% (8x10^-05^) | 57% (2x10^-04^) |
|  |  | **VAAST- Phevor** | 58% (5x10^-06^) | 61% (9x10^-04^) | 58% (8x10^-05^) |
|  |  | **CADD** | 54% (7x10^-07^) | 57% (3x10^-05^) | 50% (9x10^-07^) |
|  |  | **FATHMM** | 57% (2x10^-06^) | 52% (4x10^-06^) | 53% (4x10^-06^) |
|  | 5 - T | **Combining 5 Systems** | 71% | 71% | 71% |
|  |  | **PAVAR** | 53% (2x10^-06^) | 51% (3x10^-05^) | 50% (6x10^-05^) |
|  |  | **Exomiser v2** | 57% (1x10^-04^) | 54% (6x10^-04^) | 53% (1x10^-01^) |
|  |  | **VAAST- Phevor** | 61% (2x10^-04^) | 59% (3x10^-03^) | 59% (3x10^-03^) |
|  |  | **CADD** | 55% (2x10^-06^) | 50% (3x10^-05^) | 50% (3x10^-05^) |
|  |  | **FATHMM** | 53% (2x10^-06^) | 53% (1x10^-04^) | 53% (1x10^-04^) |
|  | FCONTROL - F | **Combining 5 Systems** | 67% | 68% | 70% |
|  |  | **PAVAR** | 57% (4x10^-03^) | 58% (6x10^-05^) | 53% (8x10^-07^) |
|  |  | **Exomiser v2** | 51% (3x10^-04^) | 58% (1x10^-03^) | 59% (4x10^-04^) |
|  |  | **VAAST- Phevor** | 59% (0.03) | 59% (8x10^-02^) | 59% (3x10^-05^) |
|  |  | **CADD** | 50% (4x10^-04^) | 56% (2x10^-04^) | 50% (3x10^-07^) |
|  |  | **FATHMM** | 53% (4x10^-04^) | 50% (6x10^-05^) | 52% (1x10^-06^) |
| **CNM** | 1 - F | **Combining 5 Systems** | 69% | 65% | 57% |
|  |  | **PAVAR** | 57% (6x10^-03^) | 51% (2x10^-02^) | 50% (0.04) |
|  |  | **Exomiser v2** | 60% (0.02) | 60% (0.32) | 52% (0.01) |
|  |  | **VAAST- Phevor** | 62% (0.08) | 57% (0.15) | 52% (0.02) |
|  |  | **CADD** | 53% (1x10^-03^) | 52% (0.02) | 53% (0.89) |
|  |  | **FATHMM** | 58% (0.01) | 51% (0.02) | 52% (0.03) |
|  | 1 - TF | **Combining 5 Systems** | 71% | 59% | 57% |
|  |  | **PAVAR** | 52% (1x10^-04^) | 51% (0.08) | 50% (0.50) |
|  |  | **Exomiser v2** | 52% (1x10-^04^) | 51% (0.08) | 51% (0.01) |
|  |  | **VAAST- Phevor** | 67% (0.39) | 59% (0.80) | 52% (0.02) |
|  |  | **CADD** | 53% (1x10^-04^) | 52% (0.09) | 53% (0.90) |
|  |  | **FATHMM** | 59% (1x10^-03^) | 51% (0.08) | 52% (0.04) |
|  | 1 - T | **Combining 5 Systems** | 73% | 57% | 57% |
|  |  | **PAVAR** | 54% (4x10^-05^) | 51% (0.19) | 51% (5x10^-02^) |
|  |  | **Exomiser v2** | 51% (9x10^-06^) | 51% (0.20) | 50% (1x10^-02^) |
|  |  | **VAAST- Phevor** | 67% (4x10^-02^) | 55% (0.61) | 52% (2x10^-02^) |
|  |  | **CADD** | 52% (8x10^-05^) | 53% (0.60) | 53% (0.91) |
|  |  | **FATHMM** | 60% (7x10^-04^) | 52% (0.32) | 52% (4x10^-02^) |
|  | FCONTROL - T | **Combining 5 Systems** | 63% | 71% | 70% |
|  |  | **PAVAR** | 53% (3x10^-03^) | 53% (5x10^-05^) | 52% (5x10^-05^) |
|  |  | **Exomiser v2** | 50% (5x10^-03^) | 61% (2x10^-02^) | 61% (6x10^-03^) |
|  |  | **VAAST- Phevor** | 60% (0.45) | 60% (1x10^-02^) | 60% (4x10^-03^) |
|  |  | **CADD** | 50% (1x10^-03^) | 54% (8x10^-05^) | 52% (1x10^-04)^ |
|  |  | **FATHMM** | 52% (2x10^-03^) | 52% (6x10^-05^) | 52% (4x10^-05^) |

**Table S4** Number of SNV obtained in 21 exome datasets according to its effect on protein sequence and position on the reference genome (GRCh37 hg19).

|  | **MD** | | **Controls** | |
| --- | --- | --- | --- | --- |
|  | **Mean ± SD** | **%** | Mean ± SD | **%** |
| Total variants | 44703 ± 7831 | 100 | 36098 ±1 2263 | 100 |
| Missense variants | 8602 ± 1034 | 19.24 | 6726 ± 2131 | 18.64 |
| Nonsense variants | 116 ± 55 | 0.26 | 75 ± 15 | 0.21 |
| Synonymous variants | 9116 ± 917 | 20.39 | 7296 ± 2417 | 20.22 |
| Intronic variants | 24977 ± 5888 | 55.87 | 20470 ± 7413 | 56.72 |
| UTR 3' or 5' variants | 1890 ± 350 | 4.23 | 1559 ± 541 | 4.32 |
| Novel variants | 3319 ± 2302 | 7.42 | 2125 ± 1063 | 5.88 |

**Table S5.** Web Resources, the URLs for software presented are as follows:

| **Web Resources** | **URLs** |
| --- | --- |
| ANNOVAR | http://www.openbioinformatics.org/annovar/ |
| Mutation Taster | http://www.mutationtaster.org/ |
| PhastCons | http://compgen.bscb.cornell.edu/phast/phastCons-HOWTO.html |
| PhyloP | http://compgen.bscb.cornell.edu/phast/help-pages/phyloP.txt |
| POLY-PHEN2 | http://genetics.bwh.harvard.edu/pph2/index.shtml |
| SIFT | http://provean.jcvi.org/genome_submit.php |
| GERP++ | http://mendel.stanford.edu/SidowLab/downloads/gerp/ |
| Exomiser v2 | https://www.sanger.ac.uk/resources/databases/exomiser/ |
| CADD | http://cadd.gs.washington.edu/ |
| FATHMM | http://fathmm.biocompute.org.uk/ |
| 1000 Genomes | http://www.1000genomes.org/dbSNP |
| NHLBI Exome Sequencing Project Exome Variant Server | <http://evs.gs.washington.edu/EVS/> |
| SHIELD: Shared Harvard Inner-Ear Laboratory Database | https://shield.hms.harvard.edu/ |
| EMBL-EBI | http://www.ebi.ac.uk/ |
| Orphanet | http://www.orpha.net/consor4.01/www/cgi-bin/OC_Exp.php?lng=EN&Expert=169189 |
| Whole exome sequencing protocols | http://www.genomics.agilent.com/en/home.jsp |
| FastQC software | http://www.bioinformatics.babraham.ac.uk/projects/fastqc/ |
| Hereditary Hearing Loss Homepage | http://hereditaryhearingloss.org/ |
| Human Phenotype Ontology database | http://human-phenotype-ontology.github.io/ |

**Table S6** Pathogenic variants scoring system (PAVAR). To calculate the score for each variant, one point was assigned for each tool which score exceed the predefined threshold.

| **NAME** | **Score = 0** | **Score = 1** |
| --- | --- | --- |
| **SIFT** | > 0.05 : tolerated | < 0.05 : deleterious |
| **Mutation Taster** | Polymorphism (p_value) | Disease causing (p_value) |
| **Grantham Matrix** | (0-50) : conservative  (51-100) : moderately conservative | (101-150) : moderately radical  (≥151) : radical |
| **PolyPhen-2** | (0.956,0.453) : possibly damaging  (0.453,0) : benign | (>0.957):probably damaging |
| **Phylop** | < 0.700 | > 0.700 |
| **PhastCons** | < 0.700 | > 0.700 |
| **GERP** | < 3.0 little evolutionary conservation | > 3.0 strong evolutionary conservation |
|  |  |  |

**Table S7:** HPO terms used to describe the AD-SNHLs.

| **HPO code** | **HPO terms** | **Number of AD-SNHLs including this HPO term** |
| --- | --- | --- |
| HP:0000407 | Sensorineural hearing impairment | 11 |
| HP:0000365 | Hearing impairment | 5 |
| HP:0000360 | Tinnitus | 5 |
| HP:0003676 | Progressive | 4 |
| HP:0005101 | High-frequency hearing impairment | 3 |
| HP:0002321 | Vertigo | 2 |
| HP:0000408 | Progressive sensorineural hearing impairment | 2 |
| HP:0008619 | Bilateral sensorineural hearing impairment | 2 |
| HP:0001730 | Progressive hearing impairment | 2 |
| HP:0008573 | Low-frequency sensorineural hearing impairment | 1 |
| HP:0000703 | Dentinogenesis imperfecta | 1 |
| HP:0011463 | Childhood onset | 1 |
| HP:0008542 | Low-frequency hearing loss | 1 |
| HP:0008615 | Adult onset sensorineural hearing impairment | 1 |
| HP:0009591 | Abnormality of the vestibulocochlear nerve | 1 |
| HP:0005102 | Cochlear degeneration | 1 |
| HP:0008596 | Postlingual sensorineural hearing impairment | 1 |
| HP:0011462 | Young adult onset | 1 |
| HP:0000405 | Conductive hearing impairment | 1 |
| HP:0004467 | Preauricular pit | 1 |
| HP:0003621 | Juvenile onset | 1 |

**Table S8** HPO terms used to describe the CNMs.

| **HPO code** | **HPO terms** | **Number of CNMs including this HPO term** |
| --- | --- | --- |
| HP:0001371 | Flexion contracture | 3 |
| HP:0001270 | Motor delay | 2 |
| HP:0000007 | Autosomal recessive inheritance | 2 |
| HP:0000508 | Ptosis | 2 |
| HP:0000218 | High palate | 2 |
| HP:0003687 | Centrally nucleated skeletal muscle fibers | 2 |
| HP:0010628 | Facial palsy | 2 |
| HP:0001284 | Areflexia | 2 |
| HP:0003677 | Slow progression | 2 |
| HP:0003236 | Elevated serum creatine phosphokinase | 2 |
| HP:0003691 | Scapular winging | 1 |
| HP:0003391 | Gowers sign | 1 |
| HP:0000602 | Ophthalmoplegia | 1 |
| HP:0002747 | Respiratory insufficiency due to muscle weakness | 1 |
| HP:0001319 | Neonatal hypotonia | 1 |
| HP:0003458 | EMG: myopathic abnormalities | 1 |
| HP:0003674 | Onset | 1 |
| HP:0001256 | Intellectual disability, mild | 1 |
| HP:0002460 | Distal muscle weakness | 1 |
| HP:0001260 | Dysarthria | 1 |
| HP:0002808 | Kyphosis | 1 |
| HP:0001618 | Dysphonia | 1 |
| HP:0002515 | Waddling gait | 1 |
| HP:0003307 | Hyperlordosis | 1 |
| HP:0003700 | Generalized amyotrophy | 1 |
| HP:0003327 | Axial muscle weakness | 1 |
| HP:0000276 | Long face | 1 |
| HP:0001761 | Pes cavus | 1 |
| HP:0001762 | Talipes equinovarus | 1 |
| HP:0002650 | Scoliosis | 1 |
| HP:0008872 | Feeding difficulties in infancy | 1 |
| HP:0000544 | External ophthalmoplegia | 1 |
| HP:0003701 | Proximal muscle weakness | 1 |
| HP:0003712 | Skeletal muscle hypertrophy | 1 |
| HP:0003388 | Easy fatigability | 1 |
| HP:0005335 | Sleepy facial expression | 1 |
| HP:0003394 | Muscle cramps | 1 |
| HP:0001324 | Muscle weakness | 1 |
| HP:0100305 | Ring fibers | 1 |
| HP:0002063 | Rigidity | 1 |
| HP:0003557 | Increased variability in muscle fiber diameter | 1 |
| HP:0003798 | Nemaline bodies | 1 |
| HP:0001374 | Congenital hip dislocation | 1 |
| HP:0003593 | Infantile onset | 1 |
| HP:0001252 | Muscular hypotonia | 1 |
| HP:0003198 | Myopathy | 1 |
| HP:0003680 | Nonprogressive | 1 |
| HP:0001380 | Ligamentous laxity | 1 |
| HP:0002905 | Hyperphosphatemia | 1 |
| HP:0001945 | Fever | 1 |
| HP:0003803 | Type 1 muscle fiber predominance | 1 |
| HP:0002913 | Myoglobinuria | 1 |
| HP:0001789 | Hydrops fetalis | 1 |

**Table S9** VAAST files. P-value of quality, no significant differences were found between WES data and the background.

| **Pedigree** | **Cases (n)** | **Controls (n)** | **p-value** |
| --- | --- | --- | --- |
| **1** | 3 | 1 | 0.872 |
| **2** | 2 | 2 | 0.409 |
| **3** | 3 | 1 | 1 |
| **4** | 3 | 0 | 0.560 |
| **5** | 3 | 3 | 1 |
